# Structural basis for difunctional mechanism of m-AMSA against African swine fever virus pP1192R

**DOI:** 10.1093/nar/gkae703

**Published:** 2024-08-21

**Authors:** Ruili Liu, Junqing Sun, Lian-Feng Li, Yingxian Cheng, Meilin Li, Lifeng Fu, Su Li, Guorui Peng, Yanjin Wang, Sheng Liu, Xiao Qu, Jiaqi Ran, Xiaomei Li, Erqi Pang, Hua-Ji Qiu, Yanli Wang, Jianxun Qi, Han Wang, George Fu Gao

**Affiliations:** College of Veterinary Medicine, Henan Agricultural University, Zhengzhou, Henan Province 450046, China; Beijing Life Science Academy, Beijing 102200, China; College of Veterinary Medicine, Shanxi Agricultural University, Jinzhong, Shanxi Province 030801, China; State Key Laboratory for Animal Disease Control and Prevention, National African Swine Fever Para-Reference Laboratory, National High-Containment Facilities for Animal Disease Control and Prevention, Harbin Veterinary Research Institute, Chinese Academy of Agricultural Sciences, Harbin Province 150069, China; College of Veterinary Medicine, Henan Agricultural University, Zhengzhou, Henan Province 450046, China; CAS Key Laboratory of Pathogen Microbiology and Immunology, Institute of Microbiology, Chinese Academy of Sciences, Beijing 100101, China; State Key Laboratory for Animal Disease Control and Prevention, National African Swine Fever Para-Reference Laboratory, National High-Containment Facilities for Animal Disease Control and Prevention, Harbin Veterinary Research Institute, Chinese Academy of Agricultural Sciences, Harbin Province 150069, China; CAS Key Laboratory of Pathogen Microbiology and Immunology, Institute of Microbiology, Chinese Academy of Sciences, Beijing 100101, China; State Key Laboratory for Animal Disease Control and Prevention, National African Swine Fever Para-Reference Laboratory, National High-Containment Facilities for Animal Disease Control and Prevention, Harbin Veterinary Research Institute, Chinese Academy of Agricultural Sciences, Harbin Province 150069, China; China/WOAH Reference Laboratory for Classical Swine Fever, China Institute of Veterinary Drug Control, Beijing 100081, China; State Key Laboratory for Animal Disease Control and Prevention, National African Swine Fever Para-Reference Laboratory, National High-Containment Facilities for Animal Disease Control and Prevention, Harbin Veterinary Research Institute, Chinese Academy of Agricultural Sciences, Harbin Province 150069, China; SUSTech Cryo-EM Centre, Southern University of Science and Technology, Shenzhen 518055, China; CAS Key Laboratory of Pathogen Microbiology and Immunology, Institute of Microbiology, Chinese Academy of Sciences, Beijing 100101, China; Department of Biological Sciences, School of life Science, Liaoning University, Shenyang, Liaoning Province 110030, China; Shanxi Academy of Advanced Research and Innovation, Taiyuan, Shanxi Province 030032, China; Shanxi Academy of Advanced Research and Innovation, Taiyuan, Shanxi Province 030032, China; State Key Laboratory for Animal Disease Control and Prevention, National African Swine Fever Para-Reference Laboratory, National High-Containment Facilities for Animal Disease Control and Prevention, Harbin Veterinary Research Institute, Chinese Academy of Agricultural Sciences, Harbin Province 150069, China; National Laboratory of Biomacromolecules, Institute of Biophysics, Chinese Academy of Sciences, Beijing 100101, China; CAS Key Laboratory of Pathogen Microbiology and Immunology, Institute of Microbiology, Chinese Academy of Sciences, Beijing 100101, China; Department of Biomedical Engineering, College of Future Technology, Peking University, Beijing 100091, China; CAS Key Laboratory of Pathogen Microbiology and Immunology, Institute of Microbiology, Chinese Academy of Sciences, Beijing 100101, China

## Abstract

The African swine fever virus (ASFV) type II topoisomerase (Topo II), pP1192R, is the only known Topo II expressed by mammalian viruses and is essential for ASFV replication in the host cytoplasm. Herein, we report the structures of pP1192R in various enzymatic stages using both X-ray crystallography and single-particle cryo-electron microscopy. Our data structurally define the pP1192R-modulated DNA topology changes. By presenting the A^2+^-like metal ion at the pre-cleavage site, the pP1192R–DNA–m-AMSA complex structure provides support for the classical two-metal mechanism in Topo II-mediated DNA cleavage and a better explanation for nucleophile formation. The unique inhibitor selectivity of pP1192R and the difunctional mechanism of pP1192R inhibition by m-AMSA highlight the specificity of viral Topo II in the poison binding site. Altogether, this study provides the information applicable to the development of a pP1192R-targeting anti-ASFV strategy.

## Graphical Abstract

**Graphical Abstract:**
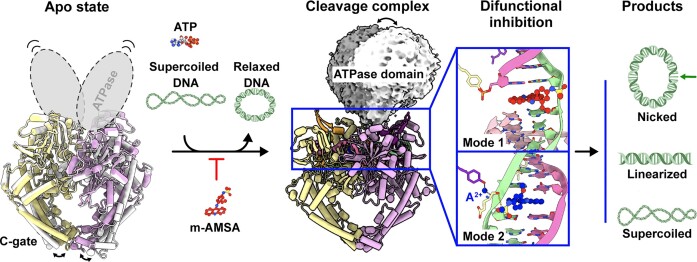

## Introduction

The African swine fever virus (ASFV) infects all members of the family *Suidae* and causes lethal African swine fever (ASF) with a high mortality rate of almost 100% (1). First found in Kenya in 1921, the ASFV has now spread globally, affecting countries in Africa, Europe, and Asia, and causing severe economic losses to the global pig industry (2). The ASFV is the only known DNA arbovirus (3) and belongs to the family *Asfarviridae*. It has been classified as a nucleo-cytoplasmic large DNA virus (NCLDV) as it possesses a large linear double-stranded DNA genome that varies in length between 170 and 190 kbp, encoding more than 150–167 open reading frames (4). The complexity of the ASFV has seriously hindered the development of safe and effective vaccines and antiviral agents against ASFV infection.

The replication cycle of ASFV is predominantly cytoplasmic, with the initial phase occurring briefly in the host cell nucleus and the bulk of DNA replication taking place in the cytoplasmic virus factory (5). As such, the virus has evolved an independent replication and transcription machinery (6) that involves a number of virus-specific enzymes such as packaging ATPases, DNA polymerases, helicases and genome-packaging related histone-like protein (7) as well as topoisomerase.

DNA topoisomerase are functional in modulating the topology of the DNA molecule in the procedures of DNA replication, transcription and chromosome segregation via creating transient breaks in the DNA (8), and are ubiquitous in archaea, bacteria, eukaryotes and viruses (9). There are two types of DNA topoisomerases (10), which differ in their ability to introduce the DNA break on one strand (type I, Topo I) or on two stands (type II, Topo II). Topo II, the main subject of this study, can be further subdivided into two subtypes that differ in sequence (8), structure and source, i.e. Topo IIA represented by Gyrase and Topo IV of bacteria and Topo II of eukaryotes, and Topo IIB of archaea (Topo VI) (11). The known viral Topo IIs all belong to Topo IIA and have been found in T4 bacteriophage, and several members of NCLDV including ASFV (12).

Benefiting from structural biology studies, the mechanism underlying the catalytic cycle of Topo II, Topo II-mediated DNA cleavage and blocking function of Topo II inhibitors have been well determined in terms of both the Gyrase (13–16), Topo IV (17, 18) and eukaryotic Topo II (19, 20). Topo IIAs exhibit a 2-fold symmetry and comprise two functional domains: an ATPase domain and a DNA binding/cleavage domain. The mechanism of enzymatic action involves the opening and closing of three gate-like dimeric interfaces, which are called the N-, DNA- and C-gate, respectively (21–23), upon ATP binding and hydrolysis and DNA cleavage that introduces a double-strand break in the DNA (G-segment) trapped in the DNA-gate for transport of another DNA strand (T-segment). It has been well established that the G-segment cleavage is related to the hydroxyl group of an active site tyrosine serving as a nucleophile and attacking the scissile phosphate of the G-segment to form a transient phosphotyrosyl bond (10); the covalent protein-DNA interaction has been termed the cleavage complex (24, 25). This nucleophilic attack relies on two metal ions and acidic amino acids in the cleavage active site (26), and has been proposed to follow a classical two-metal catalysis of DNA cleavage elucidated in depth by many nucleases and transposases, while with a novel variation in Topo II-mediated DNA cleavage (27). In classical two-metal mediated DNA cleavage, metal ion A (A^2+^) activates or assists the hydroxyl group of a catalytic water or ribose to attack the scissile DNA 5′-end, whereas metal ion B (B^2+^) interacts with and facilitates the leaving of the DNA 3′-oxyanion group (28). However, the proposed organizations of A^2+^ and B^2+^ in Topo II are different, with A^2+^ interacting with both a non-bridging oxygen of the phosphotyrosine and the DNA 3′-oxyanion group and B^2+^ located far away from the DNA 3′-oxyanion group, making the Topo II-mediated DNA cleavage process unclear (27).

Due to the irreplaceable role of topoisomerases in the life cycle, inhibitors targeting topoisomerases have achieved great success in clinical antibacterial and anticancer therapy, such as fluoroquinolones and m-AMSA, which target the bacterial and human Topo IIs, respectively. Topo II inhibitors are classified into two types based on their different mechanisms of action: poisons inhibit the Topo II via trapping or enhancing the covalent cleavage complex, while catalytic inhibitors work through various mechanisms independent of the cleavage complex, such as inhibiting DNA or ATP binding or stabilizing the non-covalent Topo II-DNA complex (29). Besides their original targets, some of the drugs were found to have a broader spectrum of inhibition. The anticancer drug etoposide was found to inhibit the gyrases of *E. coli* and *S. aureus* (30), while the m-AMSA also acts on the bacteriophage T4 Topo II (31). In ASFV, a functional viral Topo II pP1192R has been identified (32, 33), encoded by a viral gene conserved in ASFV strains, *P1192R*. pP1192R is detectable at an intermediate to late stage of ASFV infection, mainly within the cytoplasmic virus factory, and has been confirmed to be important for ASFV replication (34). Both inhibition of pP1192R by siRNA or some Topo II inhibitors reduce the titer of viral progeny (35, 36).

In this work, we presented the crystal structure of the ATPase domain of pP1192R and determined the cryo-electron microscopy (cryo-EM) structures of full-length pP1192R in multiple enzymatic activity stages, apo and DNA cleavage states, structurally defining this evolutionarily distinct Topo II and comprehensively revealing the structural basis of DNA topology change modulated by viral Topo II. pP1192R exhibited unique inhibitor selectivity, as only the eukaryotic Topo II poison m-AMSA, among the representative Topo II poisons, showed clear inhibitory activity against ASFV replication by targeting the pP1192R. Moreover, the structure of m-AMSA trapped pP1192R-DNA complex reveled a distinct difunctional mechanism of m-AMSA against pP1192R. The detailed structural information on the drug binding site of pP1192R revealed in this work open up new avenues for the design of Topo II-specific antiviral drugs. Finally, a new metal ion position that mimics the intermediate snapshot of the A^2+^ at the DNA pre-cleavage site was proposed, providing a better explanation for Topo II-mediated nucleophile formation.

## Materials and methods

### Cells and virus

Expi293F cells (ThermoFisher Scientific) were maintained in Expi293F Expression Medium (ThermoFisher Scientific) at 37 °C in 5% CO_2_ with shaking. Primary porcine alveolar macrophages (PAMs) were collected from 4-week-old specific-pathogen-free pigs and maintained in Roswell Park Memorial Institute (RPMI) 1640 Medium (Gibco) supplemented with 10% fetal bovine sera (Gibco) and 2% antibiotics-antimycotics (Gibco) at 37°C. The ASFV HLJ/2018 strain was propagated in PAMs for amplification culture. All the experiments with live virus were conducted within the animal biosafety level 3 (ABSL-3) facilities in the Harbin Veterinary Research Institute of the Chinese Academy of Agricultural Sciences.

### Protein expression and purification

To express the full-length pP1192R, the sequence of ASFV *P1192R* (Gene ID: AXB50031.1) was cloned into the plasmid pCAGGS with both N-terminal strep-tag II and the C-terminal His_6_ tag. After transient transfection and culture for 48 h, Expi293F cells were harvested and resuspended in a lysis buffer containing 10 mM 4-(2-hydroxyethyl)-1-piperazine ethanesulfonic acid (HEPES)-HCl, pH 7.4, 500 mM NaCl, 5% (vol/vol) glycerol and 1 mM PMSF. After centrifugation, the supernatant was then purified by metal affinity chromatography using HisTrap HP 5 ml column (GE Healthcare). Further purification was performed on a Superose 6 Increase 10/300 GL (GE Healthcare) size exclusion chromatography column equilibrated with 10 mM HEPES–HCl, pH 7.4, 150 mM NaCl and 5% (vol/vol) glycerol.

To express pP1192R ATPase, the coding sequence of residues 1–405 was fused to the 3′ end of the coding sequence of His_6_ tagged maltose-binding protein (MBP). A tobacco etch virus (TEV) cleavage site was inserted between the MBP and ATPase coding sequences, and a strep-tag II coding sequence was added at the 3′ end. The above sequence was cloned into pET-21a plasmid between *Nde*I and *Xho*I restriction sites. The recombinant protein was expressed in *E. coli* strain BL21 (DE3) as soluble protein by inducing the cells with 0.5 mM isopropyl-β-d-thiogalactopyranoside (IPTG) at an OD_600_ of 0.6–0.8 and expressing at 16°C. The cells were harvested after 16 h and lysed by sonication in buffer A (10 mM HEPES–HCl, pH 7.4, 150 mM NaCl and 5% (vol/vol) glycerol). After centrifugation, the supernatant was filtered with 0.22 μm membrane and loaded onto a HisTrap HP 5 ml column (GE Healthcare). The bound protein was then eluted using 500 mM imidazole dissolved in buffer A. The His-tagged MBP was subsequently removed during overnight digestion with TEV protease. The tag-free protein was then processed by passing through a 5 ml StrepTrap column (GE Healthcare). After purification on a Superdex 200 Increase 10/300 GL column (GE Healthcare), the fractions containing ATPase protein were collected and concentrated to ∼5 mg/ml for further crystal screening.

### Sedimentation velocity experiment

Sedimentation velocity experiments were performed in a ProteomeLab XL-I analytical ultracentrifuge (Beckman Coulter, Brea, CA), equipped with AN-60Ti rotor (4-holes) and conventional double-sector aluminum centerpieces of 12 mm optical path length, loaded with 380 μl of full-length pP1192R sample and 400 μl of buffer (10 mM HEPES–HCl, pH 7.4, 150 mM NaCl and 5% (vol/vol) glycerol). Before the run, the rotor was equilibrated for approximately 1h at 20°C in the centrifuge. And then experiments were carried out at 20°C and 22 000 rpm, using continuous scan mode and radial spacing of 0.003 cm. Scans were collected in 3 min intervals at 280 nm. The fitting of absorbance versus cell radius data was performed using SEDFIT software (https://sedfitsedphat.nibib.nih.gov/software/default.aspx) and continuous sedimentation coefficient distribution c(s) model, covering range of 0–25 S. Biophysical parameters of the buffer: density ρ = 1.0202 g/cm^3^, viscosity η = 0.011916, and proteins: partial specific volume *V*-bar = 0.73000 cm^3^/g.

### Crystallization, data collection and structure determination

For crystallization, AMP-PNP was added to the pP1192R-ATPase protein to a final concentration of 2 mM and the mixture was incubated overnight. All crystals were generated utilizing the sitting drop vapor diffusion method with 1 μl protein mixed with 1 μl reservoir solution. This mixture was then sealed and equilibrated against 100 μl reservoir solution at 16°C. High resolution pP1192R-ATPase crystals were grown in 0.2-M lithium acetate dihydrate, pH 7.9 and 20% (w/v) PEG 3350 within a week. Crystals were harvested by transferring them into their respective reservoir solutions, supplemented with 20% (vol/vol) glycerol, before being looped and rapidly frozen in liquid nitrogen for subsequent data collection. The data was collected at Shanghai Synchrotron Radiation Facility (SSRF) BL02U1 (wavelength, 0.973387 Å).

The dataset was processed using HKL2000 software (37). The structure of the pP1192R-ATPase was determined by molecular replacement method using PHASER (38) with the structure of *S. cerevisiae* Topo II ATPase domain (PDB code 4GFH) (39) as the search model. Atomic models were completed using Coot (40) and refined with phenix.refine of the Phenix software suite (41). The stereochemical quality of the final models was assessed through MolProbity (42). Detailed statistics regarding data collection, processing, and refinement are presented in Supplementary Table 2. Structural figures were generated using PyMOL (http://www.pymol.org), unless otherwise specified.

### Nucleic acid preparation

The sequence of the duplex DNA substrate was selected based on findings from Strumberg et al., which highlighted drug-specific base preferences near topoisomerase II cleavage sites (43). In the case of m-AMSA, adenine (A) at position +1 was favored. Therefore, we searched the genome of the virus strain, from which the protein sequence in the study was derived, for a sequence with an adenine (A) at position +1 and a thymine (T) at position +4 to ensure that the reverse DNA strand would also have an adenine (A) at position +1. Upon identification of a suitable sequence meeting these criteria, the nucleotide sequence from +1 to +12 was determined. Subsequent adjustments were made to the sequence from –1 to –8 to optimize the GC ratio of the entire sequence and the final sequence (5′-CCGTGAATAGCTATCCAATC-3′ and 5′-GATTGGATAGCTATTCACGG-3′) were determined.

The DNA oligonucleotides were dissolved in a solution containing 10 mM HEPES–HCl (pH 7.4) and 150 mM NaCl. To form the double-stranded DNA, two strands were mixed in a 1:1 molar ratio and annealed by incubating at 95°C for 5 min, then gradually decreasing the temperature by 1°C every minute until it reached 20°C.

### Cryo-EM sample preparation and data acquisition

For pP1192R-DNA-m-AMSA complex, purified full-length pP1192R protein was mixed with the 20 bp dsDNA at a 1:1 molar ratio with a final protein and DNA concentration of 2.8 μM. The m-AMSA (Selleckchem) dissolved in DMSO and MgCl_2_ was added into the protein-DNA mixture to reach a final concentration of 0.5 and 5 mM, respectively. The mixture was then incubated at 30°C for 37 min.

The cryo-samples of both apo pP1192R and pP1192R–DNA–m-AMSA complex were vitrified using a Vitrobot Mark IV (ThermoFisher Scientific) plunge freezing device. Specifically, 4.0 μl of pP1192R–DNA–m-AMSA complex at 1.0 mg/ml was applied to an Au Quantifoil 1.2/1.3 holey carbon grid that had been glow discharged for 25 s, while 4.0 μl of apo pP1192R at 0.1 mg/ml was applied to a graphene oxide (GO) coated grids (R1.2/1.3 300 mesh). The above grids were then blotted under different conditions, i.e. blotting time 4 s with blotting force 0 for the pP1192R–DNA–m-AMSA complex and blotting time 2 s with blotting force 0 for the apo pP1192R, at a temperature of 4°C and a humidity level of >99%. The grids were then plunged in liquid ethane for freezing.

For data collection, the prepared grids were transferred to a 300 kV Titan Krios transmission electron microscope equipped with Gatan K3 detector and GIF Quantum energy filter. Movies were collected at 105 000× magnification with a calibrated pixel size of 0.85 Å over a defocus range of –1.0 μm to –2.0 μm in super resolution counting mode with a total dose of 60 e^–^/Å^2^ using EPU (ThermoFisher Scientific) automated acquisition software for the pP1192R–DNA–m-AMSA complex sample. Movies were collected at 64 000× magnification with a calibrated pixel size of 1.08 Å over a defocus range of –1.0 to –2.0 μm in super resolution counting mode with a total dose of 50 e^–^/Å^2^ using EPU automated acquisition software for apo pP1192R sample.

### Image processing

The detailed data processing workflow is summarized in Supplementary Figure 1 and Supplementary Figure 6. All the raw dose-fractionated images stacks were 2× binned, aligned, dose-weighted and summed using MotionCor2 (44). The contrast transfer function (CTF) estimation, particle picking and extraction, 2D classification, *ab initio* model generation, 3D refinements were performed in cryoSPARC v.4.2.0 (45).

For the pP1192R–DNA–m-AMSA complex, a total of 4530 micrographs were collected for this dataset. Particles were picked using the blob-pick procedure in cryoSPARC from 500 micrographs, which were then subjected to 2D classification. After three rounds of 2D classification, high-quality particles with different views were selected for Topaz (46) training to generate the Topaz model. A total of 1957481 initial particles were picked from entire micrographs using the Topaz procedure and extracted with a box size of 360 pixels. Following extensive 2D classification, junk particles and contaminations were removed and approximately 459 528 particles remained for heterogeneous refinement. Among the six resulted classes, two classes displayed clear features of secondary structural elements and were selected for non-uniform refinement which yielded a density map at 2.79 Å resolution. To analyze whether there were different conformations on the DNA binding/cleavage domain, we generated a mask on this region and performed 3D classification. However, all classes contain DNA substrate with only single-strand breaks (SSB). Based on this, we combined all approximately 271 076 particles for local refinement to improve the local resolution of the DNA binding/cleavage domain. A final round of local refinement was performed and generated a final map at 2.76 Å resolution. To improve the local resolution of the ATPase domain, we generated a mask around the ATPase domain and performed 3D classification without alignment to pick out good particles with complete ATPase density. Two distinct classes were obtained after the 3D classification and subjected to non-uniform refinement which generated the EM density maps at resolutions of 3.23 and 3.17 Å, respectively. The final map was sharpened using DeepEMhancer (47).

For the apo pP1192R dataset, the image processing was similar. A total of 620 515 initial particles were picked and extracted from 2134 micrographs. After extensive 2D classification, approximately 286 450 good particles were selected for heterogeneous refinement. Among three classes, one dominant class contained 72.91% of the total particles and displayed clear features of secondary structural elements. These particles were then subjected to 3D classification which generated two different conformations representing the closed and the open states. The two states were processed with non-uniform refinement separately, yielding final density maps at 2.98 resolution and 2.77 Å resolution, respectively. The final map was sharpened by DeepEMhancer.

### Model building and structure refinement

The pP1192R–DNA–m-AMSA complex (*S. cerevisiae* Topo II, PDB 4GFH), apo pP1192R in the open state (human Topo IIα in state 2, PDB 6ZY5) and apo pP1192R in the closed state (human Topo IIα in state 1, PDB 6ZY6) were rigidly docked into the density map using Chimera (48), Mutation and manual adjustment were carried out with Coot v.0.9.3 (49). Glycans were added at N-linked glycosylation sites in Coot. Each residue was manually checked with the chemical properties taken into consideration during model building. Several rounds of the real-space refinement in Phenix-1.20.1(41) and manually building in Coot were performed until the final reliable models were obtained. Molprobity (42) was used to validate geometry and check structure quality. Statistics associated with data collection, 3D reconstruction and model building were summarized in Supplementary Table 1. Figures were generated using ChimeraX (50) and PyMOL (http://www.pymol.org).

### Relaxation assay

The relaxation activity was assessed using negatively supercoiled pUC19 plasmid DNA in a reaction buffer consisting of 50 mM Tris–HCl (pH 7.4), 75 mM NaCl, 5 mM MgCl_2_, 2 mM ATP and 1 mM DTT. The inhibitors were first dissolved in 100% DMSO to produce a series of 10-fold stock solutions. Reactions were then set up in a 10 μl reaction volume, where pP1192R (240 nM) or *Acinetobacter baumannii* topoisomerase IV (240 nM) or human Topo IIα (15 nM) was incubated with 250 ng pUC19 in the presence of 1 μl of inhibitor stock solutions at various concentrations. For the mock group, 1 μl of DMSO was added to maintain the same concentration as in the drug-treated groups. The incubation was conducted at 37°C for 30 min and was terminated by adding 1% (w/v) SDS and 12.5 mM EDTA. Subsequently, 2.5 μl of 5× running buffer, which consists of 5% SDS, 0.125% bromophenol blue and 25% glycerol, was added into the reaction. The reaction was then separated through electrophoresis on a 1% (w/v) agarose gel at 80 V for 3 h.

### Cleavage assays

The reactions were carried out under the same conditions as those in the relaxation assay. Following a 30-min incubation, 1% (w/v) SDS, 12.5 mM EDTA and 0.2 mg/ml of proteinase K were added, followed by an additional incubation at 42°C for 60 min. To completely terminate the reaction, we introduced a stop solution, which consisted of a 5× buffer comprising 5% SDS, 0.125% bromophenol blue and 25% glycerol. Subsequently, the reaction mixture was separated through electrophoresis on a 1% (w/v) agarose gel at 80 V for 3 h.

### Cell viability assay

The cytotoxicity assay of m-AMSA on PAMs was evaluated by cell counting kit 8 (CCK-8) according to the manufacturer's protocols. In brief, PAMs were seeded into a 96-well plate and cultured for 24 h. Serially diluted compounds or DMSO (control) were added to treat the cells for an additional 24 and 48 h, respectively. After incubation, the medium was removed and the CCK-8 solution was added to each well and incubated for 1 h at 37°C. The absorbance of each well was measured at 450 nm using a Microplate Reader (PE, Multiscan Spectrum Enspire) and then plotted using GraphPad Prism 8 software.

### In vitro antiviral assays

PAM cells were seeded into 24-well plates at a density of 5 × 10^5^ cell/well. The compounds were 2-fold diluted ranging from 10 μM to 0.3125 μM before being incubated with an equal volume of ASFV at a multiplicity of infection (MOI) of 0.1, and the mixtures were incubated for 1 h at 37°C. After removal of the supernatants, cells were washed twice with phosphate-buffered saline (PBS) and incubated with compounds containing medium at the indicated concentrations for 24 and 48 h, respectively. The supernatants of each well were collected, and the genome copies and the viral titers of ASFV were determined by quantitative real-time PCR (qPCR) and HAD_50_ assay, respectively.

For qPCR, the ASFV genomic DNA in the cells supernatants was extracted using the MagaBio Plus virus DNA purification kit (BioFlux) according to the manufacturer's protocols. The ASFV genomic copies were detected by qPCR on the QuantStudio system (Applied Biosystems, USA) based on a previously described method (51).

For HAD_50_ assay, PAM cells (5 × 10^4^) were seeded into 96-well plate, and infected with 10-fold serially diluted ASFV. Then, the porcine red blood cells (5 × 10^5^) were added to each well, and the ‘rosettes’ of red blood cells was observed on the fifth day using an optical microscope. The HAD_50_ (50% hemadsorption doses per ml, HAD_50_/ml) were calculated using the method of Reed and Muench (52).

### Comet assay

The comet assay was performed using a comet assay kit (Biogradetach). In brief, PAMs (2 × 10^6^) infected with ASFV (MOI = 1) were exposed to m-AMSA (100 μg/ml) from 16 to 18 hpi. Controls included infected cells not exposed to m-AMSA and non-infected cells exposed to m-AMSA. At the indicated time points, cells were collected and resuspended at 5 × 10^5^ cells/ml in ice-cold PBS. Cells were combined with low melting point agarose at a 1:10 ratio (v/v), mixed well by pipetting, and immediately 75 μl/well were transferred onto frosted comet slides pre-coated with a base layer of 1% normal melting point agarose. The slides were transferred to a dark room for 15 min at 4°C. The comet slides were then immersed in a pre-chilled alkaline lysis solution (2.5 M NaCl, 100 mM EDTA, 10 mM Tris–HCl, 1% Triton X-100, pH 10) in a dark room for 1 h at 4°C. Before electrophoresis, the slides were incubated in a fresh cold electrophoresis buffer (1 mM EDTA, 300 mM NaOH) for 30 min at 4°C in the dark to allow DNA unwinding. Electrophoresis was performed at 19 V on ice for 30 min. After electrophoresis, the comet slides were horizontally transferred to a container with pre-cooled 0.4 mM Tris–HCl (pH 7.5) buffer and neutralized three times for 10 min each at 4°C. The buffer was then discarded, and 50 μl/well of 1 × PI dye was added for staining in the dark at room temperature for 10 min. After one PBS wash, the comet slides were analyzed and recorded using a fluorescence microscope (EVOS M5000, ThermoFisher).

## Results

### Cryo-EM reconstruction of apo pP1192R reveals two conformations of the DNA-binding/cleavage domain

**Figure 1.**
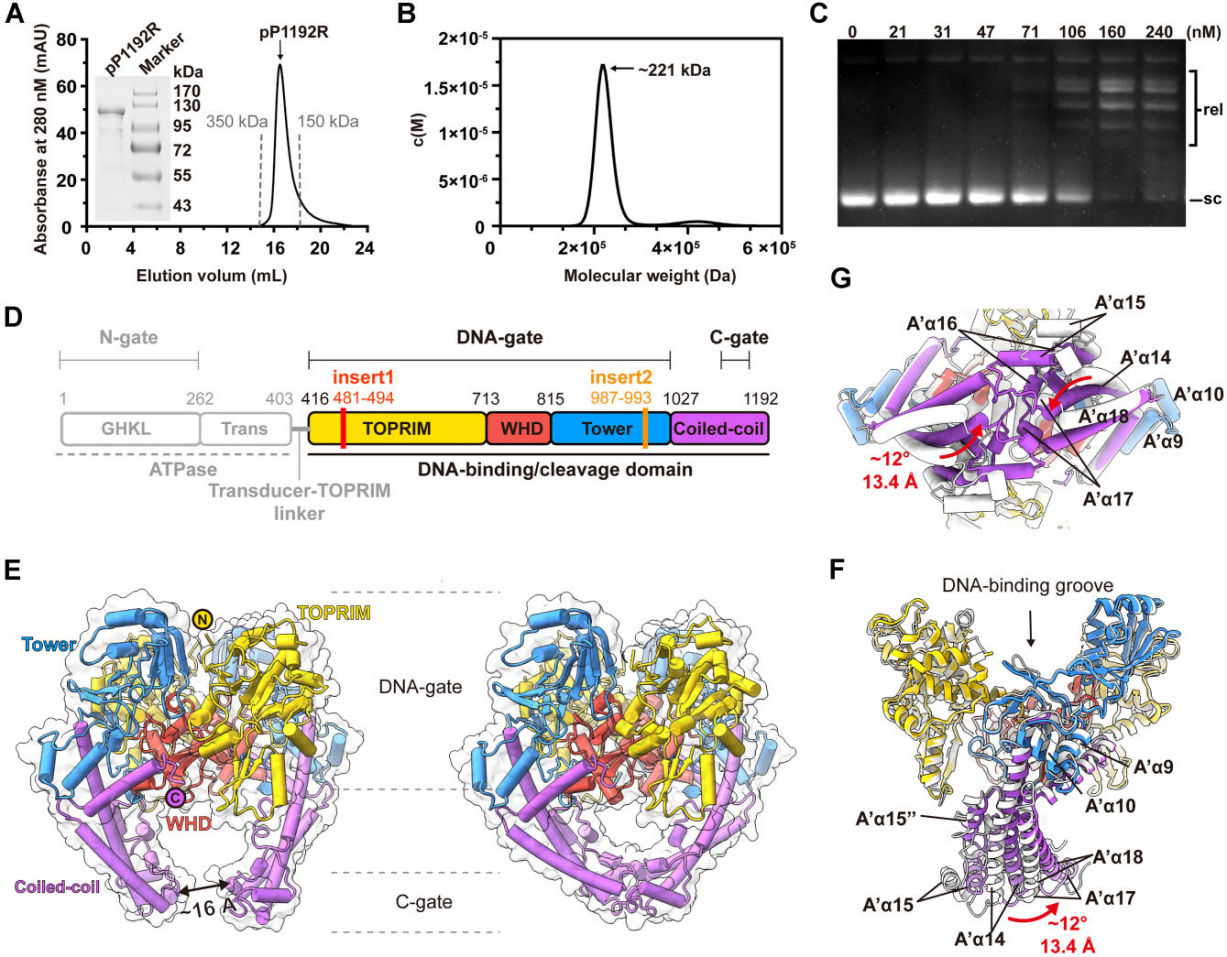
Biochemical characteristics and overall architectures of ASFV pP1192R in the apo state. (**A**) The gel filtration profile of pP1192R protein is shown in black, with gray dotted lines marking the elution peaks of two standard proteins. (**B**) Sedimentation velocity profile of pP1192R. Sedimentation coefficient distributions c(M) calculated from the sedimentation velocity profile with the calculated molecular weight shown. (**C**) Relaxation assays catalyzed by pP1192R. pP1192R was tested at increasing concentrations as indicated above each lane. Relaxed (rel) and supercoiled (sc) forms of DNA are indicated. (**D**) The schematic representation of pP1192R. Functional domains, dimerization gates, and sequence insertions are labeled. The domain organization of the DNA binding/cleavage domain is highlighted. (**E**) The overall structures of dimeric pP1192R DNA binding/cleavage domain, featuring its C-gate in either the open (left) or closed (right) state. Each domain shown in the cartoon representation is colored as in (D) and the surface representation is colored white. (**F** and **G**) Conformational changes of the pP1192R C-gate between open (white) and closed (colored as shown in (D)). The secondary structural elements are labelled according to Supplementary Figure 2C.

The full-length pP1192R was produced in mammalian cells and subsequently purified for enzymatic and structural analysis. The recombinantly expressed pP1192R protein is homogeneously dimeric with a molecular weight of about 221 kDa, as determined by both gel-filtration chromatography and analytical ultracentrifugation (Figure 1A, B). The enzymatic activity of this purified pP1192R protein was then confirmed by relaxation assay (Figure 1C).

To capture the intact structure of full-length pP1192R, the protein sample was then analyzed by single-particle cryo-EM. After collecting and processing 2134 cryo-EM images, two types of particles, referred to as State 1 and State 2, were identified and reconstructed to the overall resolution of 2.77 and 2.98 Å with 131 161 and 78 195 particles, respectively (Supplementary Table 1 and Supplementary Figure 1). The electron density was only observable for the DNA binding/cleavage domain in both states, while the electron density of the N-terminal ATPase domain was absent.

Following the process of atomic model building based on the EM density map, the apo structure of the pP1192R DNA binding/cleavage domain was well resolved in both states, with the C-terminus of the protein (C-gate) adopting distinct conformations, accordingly described as ‘open’ (State 1) and ‘closed’ (State 2). These apo structures can be traced for 779 residues, ranging from D416 to H1192 (Figure 1D, E), except for the region containing residues G471-N501. Despite pP1192R is highly divergent from other prokaryotic or eukaryotic Topo IIs based on the phylogenetic analysis (32), the DNA binding/cleavage domain of pP1192R presents high similarity with other Topo IIAs in terms of the overall dimeric configuration as well as the subdomain assembly characteristics (13, 20, 53) (Figure 1E, Supplementary Figure 2).

The pP1192R is devoid of the variable C-terminal domain (CTD), which is commonly present and contribute to the enzyme's catalytic activities in other Topo IIAs homologs (13). The other typical domains of the DNA binding/cleavage core characterized in Topo IIAs, that is, the metal-binding topoisomerase/primase (TOPRIM) domain, the catalytic tyrosine (Y800 in pP1192R)-containing winged helix domain (WHD), the Tower domain, and the coiled-coil helices, are well illustrated in the two pP1192R apo structures (Figure 1E, Supplementary Figure 2). As described for other Topo IIAs, the TOPRIM and Tower domains from two protomers interact to build the sidewall of the DNA-binding groove, and the two WHD domains bind together via their respective A' α3 and A' α4 helices to form the base of the DNA-binding groove (Supplementary Figure 3A). In the closed state, an extra dimeric interface, the C-gate, was formed by the interaction between the coiled-coil helices of two protomers, which retains a distance of ∼16 Å in the open state (Figure 1E). No significant conformational changes were observed in the DNA-binding groove when comparing the two apo states (Supplementary Figure 3A), while the coiled-coil helices underwent a distinct conformational rearrangement, presenting the dynamic cycling of the C-gate between open to closed (Figure 1F, G). The coiled-coil regions (from A' α14 to A' α18) of each protomer move towards one another in the closed state, with the furthest end moving a distance of ∼13.4 Å and a reorientation of ∼12° relative to the open state. These two structures superimposed well with the recently reported apo structures of pP1192R derived from the genotype II ASFV (Georgia07) (9) or the genotype I ASFV (L60) (54) despite 27 or 28 amino acids differences in the DNA-binding/cleavage domain (Supplementary Figure 3B-D). These residues are all located away from the DNA binding or cleavage site, consistent with the functional conservation of pP1192R.

Sequence alignment and structure superimposition between representative Topo II homologs identified the detailed features within each domain of pP1192R (Supplementary Figure 2). First, extra amino acids (insert1) were detected exclusively in pP1192R within the TOPRIM domain, involving residues V481-V494. In the apo structures, this region together with the flanking residues (G471-N501) were missing in the EM density map, whereas in other Topo IIAs this region is well presented as a loop involved in DNA binding. Second, in both pP1192R and the prokaryotic Topo IIAs, a distinct A’α10’ helix was shown to be located at the periphery of a evolutionarily conserved A’α10 helix and to interact with the A’α10 helix and the A’α14 helix of the coiled-coil domain. In addition, two structural features were found in pP1192R that are positioned at the DNA binding interface of the TOPRIM domain, i.e. a relatively short linkage (A’α11’ helix) between the A’α11 helix and B’β12 strand and a longer loop introduced by a seven amino acid insertion (insert2) bridging the A’α12 and A’α13 helices. Finally, compared to the conservation of the WHDs, the greatest sequence variability in DNA binding/cleavage domain was found at the region between the A’α15 and A’α16 helices of the coiled-coil region. In pP1192R, this region is well rebuilt as two helices, designated A’α15’ and A’α15’’, respectively. These two helices bind tightly to the A’α14 helix, while the connecting loop of the two helices points towards the base of the Tower domain and is stabilized by the interaction with the A’α8 helix.

### m-AMSA prevents ASFV replication by targeting the pP1192R

**Figure 2.**
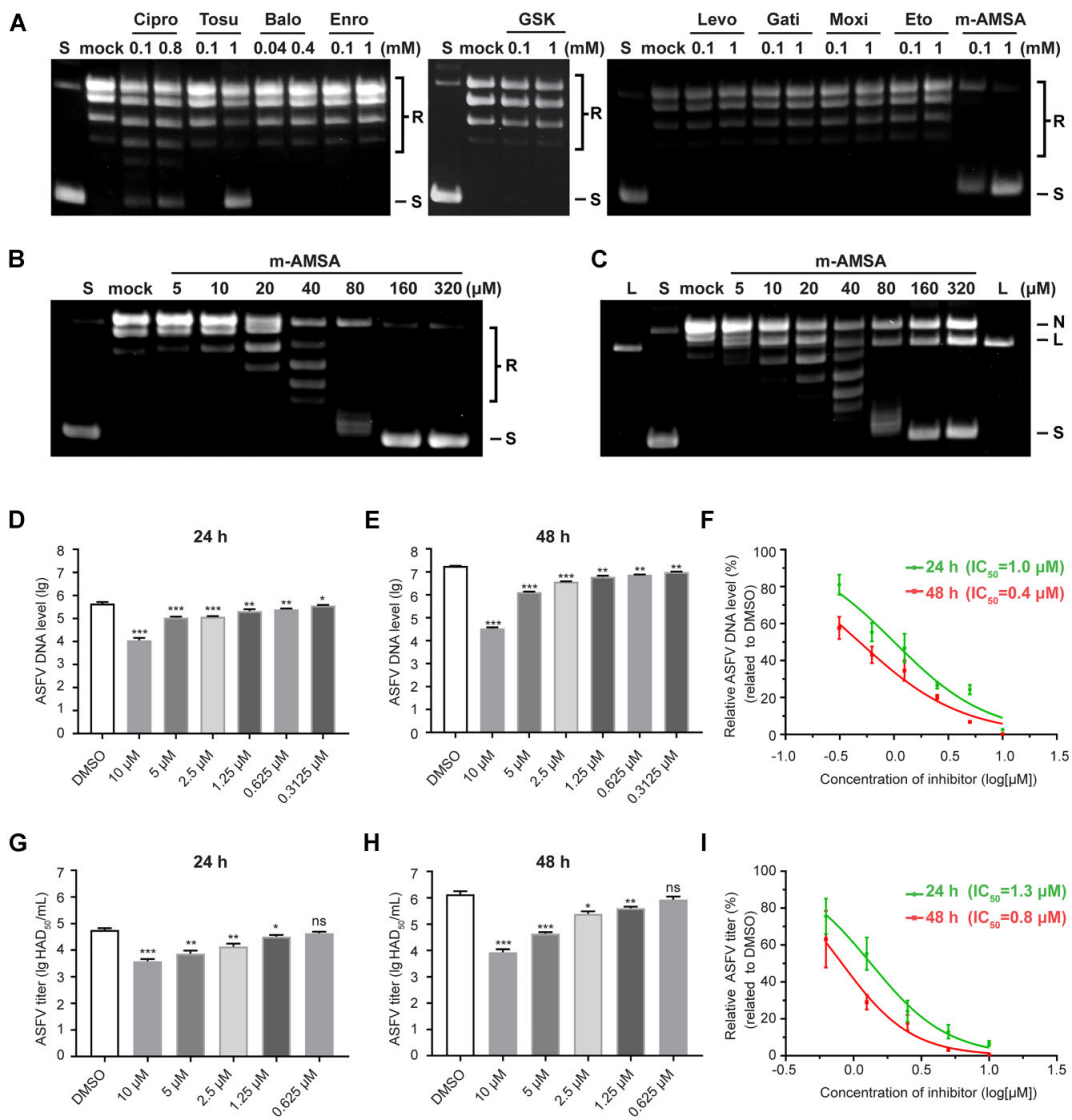
Evaluation of representative Topo II poisons in pP1192R inhibition and the effect of m-AMSA on ASFV replication. (**A** and **B**) Evaluation of representative Topo II inhibitors in pP1192R blockade by relaxation assays. The final concentrations of drugs are shown. For the initial experiment (A), certain drugs were not evaluated at 1 mM due to solubility constraints and were instead tested at their highest concentration without drug precipitation. Fluoroquinolones are all abbreviated by omitting the suffix ‘floxacin’. The complete designations for GSK and Eto are GSK299423 and etoposide, respectively. S and R, supercoiled and relaxed DNA gel markers, respectively. Mock represents the control experiments containing the same volume of DMSO instead of drugs. (**C**) The formation of the cleaved complex induced by m-AMSA is assessed by the cleavage assay. The final concentrations of drugs are shown. Linear (L), nicked (N) and supercoiled (S) forms of DNA are labelled. Mock represents the control experiments containing the same volume of DMSO instead of drugs. (D–F) Effect of m-AMSA on virus replication evaluated by ASFV DNA levels. The p72 gene level (*n* = 6) after 24 h (**D**) or 48 h (**E**) treatment with m-AMSA is assessed by qPCR and the IC_50_s are calculated (**F**). Data were compared to DMSO. (G–I) Effect of m-AMSA on infectious virion yield evaluated by HAD assays. The yield of infectious virions (*n* = 3) after 24 h (**G**) or 48 h (**H**) treatment with m-AMSA is evaluated by HAD assays and the HAD_50_s are calculated (**I**). Data were compared to DMSO. Data indicate the mean ± s.d. P values were analyzed with two-tailed unpaired t test (NS, not significant, *P* > 0.05; ∗, *P*< 0.05; ∗∗, *P* < 0.01; ∗∗∗, *P* < 0.001).

Inspired by the overall configuration similarity of pP1192R to other Topo IIAs, we next investigated the ability of some Topo IIA inhibitors to prevent pP1192R function at the protein level. Seven representative 2nd, 3rd and 4th generation fluoroquinolones and a new class bacterial topoisomerase inhibitor (NBTI) poisoning prokaryotic Topo IIs as well as two anticancer drugs that target eukaryotic Topo IIs were first evaluated in relaxation assays in the presence of pP1192R or their responding targets together with the supercoiled pUC19 plasmid. Among the fluoroquinolones, only ciprofloxacin and tosufloxacin showed some of inhibitory activity against pP1192R (Figure 2A). In contrast to their significant potency against prokaryotic Topo IIAs (Supplementary Figure 4), these two compounds failed to completely counteract the relaxation function of pP1192R even at exceedingly high concentrations (up to 0.8 or 3 mM, respectively) (Supplementary Figure 4). Among the eukaryotic Topo II poisons, m-AMSA showed a marked inhibitory effect on pP1192R in a dose-dependent manner and can completely counteract the relaxation function of pP1192R at high concentrations (Figure 2B). DNA cleavage assays were then performed to further clarify the inhibitory mechanism of ciprofloxacin, tosufloxacin and m-AMSA against pP11192R. Surprisingly, m-AMSA, but not ciprofloxacin and tosufloxacin, induced both nicked and linear DNA products that increased with drug concentration, indicating a combined working mode of m-AMSA against pP1192R, i.e. generation of either single-strand break (SSB) or double-strand break (DSB) of the DNA substrate (Figure 2C and Supplementary Figure 4).

Due to the essential role of pP1192R in ASFV replication (35) and with the evidence of the inhibitory effect of m-AMSA against pP1192R, we further tested whether m-AMSA can inhibit ASFV replication in porcine alveolar macrophages (PAMs). The cytotoxicity of m-AMSA to PAMs was first tested by a cell viability assay (Supplementary Figure 5). m-AMSA suppressed PAMs proliferation when used at concentrations >10 μM, with a 50% cytotoxic concentration (CC_50_) of about 23.6 μM after 24 h of treatment, while the CC_50_ decreased to ∼16.8 μM after 48 h of treatment. We then selected 10 μM as the optimal non-toxic concentration for m-AMSA in the subsequent antiviral effect analysis, since at this concentration there is only weak (12.7% after 48 h exposure) or no (for 24 h exposure) cell growth inhibition. The effect of m-AMSA on viral production was tested by both real-time qPCR targeting p72 gene and hemadsorption (HAD) assay. Notably, m-AMSA displayed strong inhibition of ASFV DNA levels in a dose-dependent manner, with a reduction of 97.4% and 99.8% (related to DMSO), respectively, after 24 and 48 h of drug treatment at concentrations of 10 μM compared to that in cells infected with virus alone (Figure 2D–F). The inhibitory concentration to reduce 50% of virus production (IC_50_) was estimated to be 1 or 0.4 μM after 24 or 48 h drug treatment, respectively (Supplementary Figure 5). The further HAD assay, which reflects the adsorption of red blood cells on the surface of ASFV-infected macrophages by identifying the characteristic ‘rosette’ formation, provided a comparable result. The titer of infectious progeny ASFV particles in the supernatants were reduced by m-AMSA in a dose-dependent manner, with the highest reduction from 5.1 lg HAD_50_/ml to 3.6 lg HAD_50_/ml (93.3% related to DMSO) or from 6.6 lg HAD_50_/ml to 4 lg HAD_50_/ml (99.3% related to DMSO) after 24 or 48 h of 10 μM drug treatment, respectively (Figure 2G–I).

Since m-AMSA is also a potent poison of eukaryotic Topo IIs, a single cell electrophoresis analysis (comet assay) was then performed to clarify the targets of the m-AMSA in its antiviral effects (Supplementary Figure 5D–F). PAM cells infected with ASFV (MOI = 1) were exposed to m-AMSA (100 μg/ml) from 16 hpi to 18 hpi. The PAM cells infected and not exposed to m-AMSA (Supplementary Figure 5D) and the PAM cells non-infected and exposed to m-AMSA (Supplementary Figure 5E) were used as controls. No comet was identified in the infected and non-exposed group, indicating that ASFV infection alone cannot induce detectable DNA fragmentation. Although the drug concentration we used in the antiviral assay has little or no effect on cell growth, fragmented DNA was still observed in the non-infected and exposed group (Supplementary Figure 5E), indicating the involvement of host Topo II in m-AMSA-induced DNA fragmentation. However, it is apparently to a lesser extent than that in the infected and exposed group (Supplementary Figure 5F). This suggests that m-AMSA impacts ASFV replication through combined effects, i.e. interfering with the activity of both the ASFV pP1192R (direct inhibition) and host PAM cells (indirect inhibition).

### Overall architecture of full-length pP1192R in complex with DNA trapped by m-AMSA

**Figure 3.**
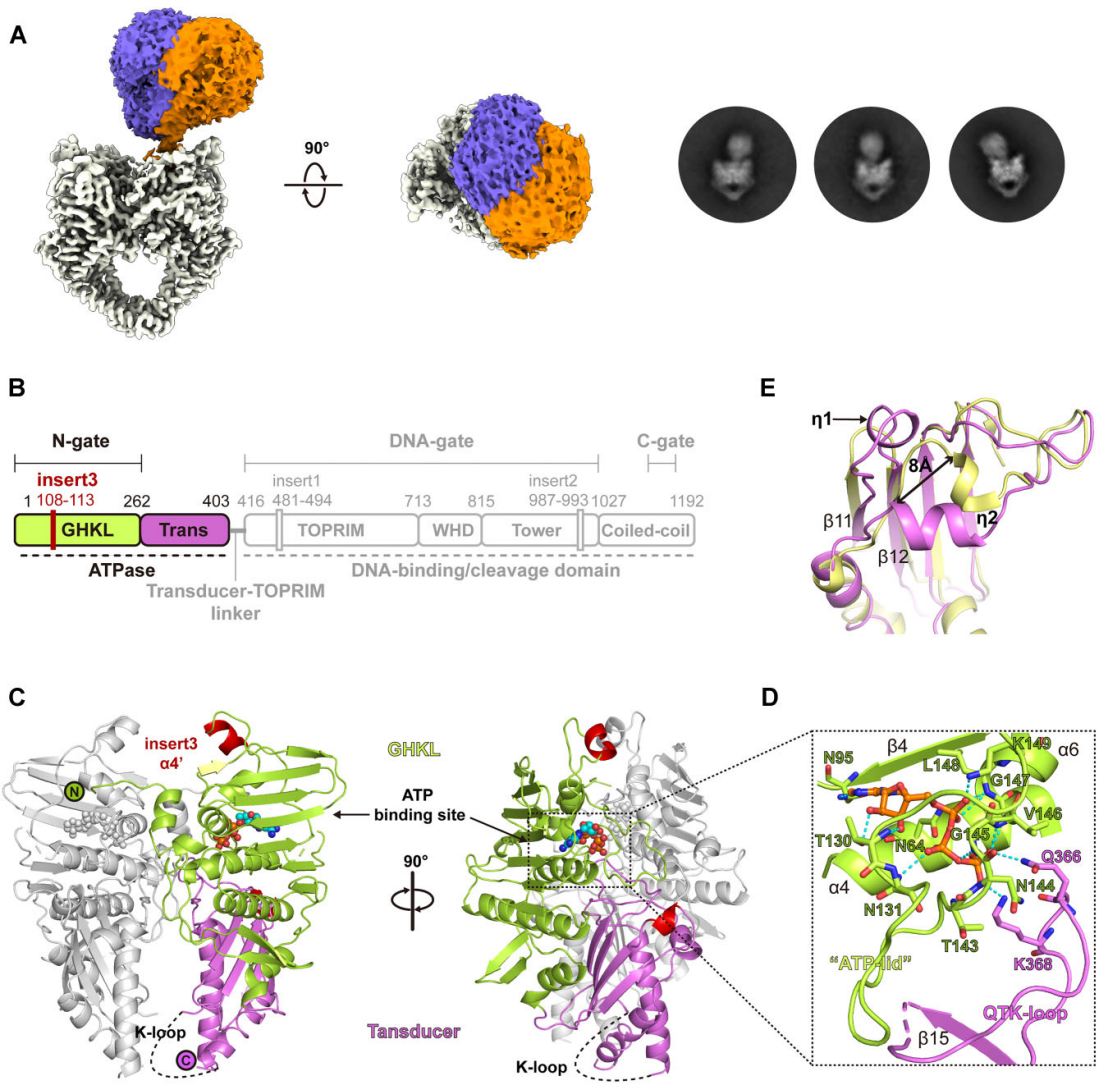
Structure of the pP1192R ATPase domain. (**A**) 3D and 2D classification demonstrates the flexibility of the ATPase domain relative to the DNA-binding/cleavage domain. (**B**) The schematic representation of pP1192R. Functional domains, dimerization gates and sequence insertions are labeled. The domain organization of the ATPase domain is highlighted. (**C**) The dimeric structure of the ATPase domain of pP1192R. The domains in one protomer are colored as in (B), while the other protomer is colored gray. The α4’ helix (insert3) and η1 helix are highlighted in red. The ATP binding site is indicated by a dashed square. (**D**) Close-up view of the ATP binding site. Amino acids involved in hydrogen bonds and salt bridges with the AMP-PNP are shown as sticks and labelled. (**E**) Structural superimposition of the ATPase domains in pP1192R (yellow) and *S. cerevisiae* Topo II (purple) reveals the distinct conformation of the η2 helix in pP1192R ATPase domain.

The differential inhibitor selectivity of pP1192R suggests the specificity of this evolutionarily distinct viral Topo II that call for a further understanding of the atomic details of pP1192R in terms of its working state and the drug binding site. To this end, the cryo-EM sample of DNA-bound pP1192R was prepared by adding both a DNA duplex, m-AMSA, and AMP-PNP. Consistent with the structures of its homologs (13), the dimerized ATPase domain of pP1192R showed flexibility and displayed different orientations relative to the DNA binding/cleavage domains in the 2D classification (Supplementary Figure 6 and Figure 3A). An *ab-initio* 3D model was calculated with 271 076 particles and the global architecture of full-length pP1192R in complex with DNA substrate, inhibitor and AMP-PNP was resolved. To get information on the flexible ATPase region, a 3D-focused classification was performed on this region yielding two classes of particles which was refined to 6–9 Å local resolution of the ATPase region (Supplementary Table 1 and Supplementary Figures 6 and 7). Despite the low resolution, these two structures revealed the orthogonal orientation of the ATPase over the DNA binding/cleavage domain of pP1192R with an obvious swing (Figure 3A).

### Structure of the pP1192R ATPase domain

To provide detailed information of the ATPase domain and support the model building of the full-length pP1192R, the atomic resolution structure of the ATPase domain of pP1192R in complex with the nonhydrolyzable homolog of ATP (AMP-PNP) was determined by X-ray crystallography at 1.8 Å using a truncated pP1192R-ATPase protein expressed as soluble protein in *E. coli* cells. The final refined model has reasonable R-factors and stereochemistry (Supplementary Table 2). The electron density map is of good quality, with only a few flexible terminal residues and two regions containing R70-T76 and I334-R343 being disordered. Interestingly, the region R70-T76 was found to exhibit large conformational changes under different redox conditions, as detailed by Chang et al. (54), involving the formation and breakage of the disulfide bond between C72 and C138, which may reflect a redox regulatory mechanism of pP1192R. Our structure shows high overall similarity to both the reduced and oxidized forms, with the RMSD values of 0.18 and 0.102 Å, respectively (Supplementary Figure 8A). The observed flexibility in this region of our structure may reflect an intermediate state that transitions to the reduced or oxidized conformation.

Structural comparison using the Dali server (55) against the full Protein Data Bank (PDB) of known structures revealed that the pP1192R-ATPase shares great structural similarities with both its eukaryotic and prokaryotic homologs, while most resembles the ATPase domains of the eukaryotic Topo IIAs represented by *S. cerevisiae* (56) (*Z* score = 42, r.m.s.d. of 2.0 Å over 352 aligned Cα residues). The pP1192R-ATPase displayed a conserved heart-shaped dimeric architecture as its homologs (56) with each protomer consisting of two well characterized regions, i.e. the N-terminal GHKL ATP-binding fold and the C-terminal Transducer domain (Figure 3B, C). Structural comparison of the ATPases within the representative Topo IIAs revealed that the typical secondary structural elements (eight antiparallel β-strands plus four α-helices in the GHKL fold and four β-strands plus two α-helices in the transducer domain) in these two regions were conserved in pP1192R (Supplementary Figure 8B). The amino acid insertion specific for eukaryotic Topo IIAs (β1 and β2 strands) (56) is also present in pP1192R with a corresponding conformation. The bound AMP-PNP in the pP1192R ATPase adopts an identical orientation to that of the other Topo IIAs ATPases (Supplementary Figure 8C), and the secondary structure elements comprising the binding pocket are conserved (Figure 3D). The primary interactions were correspondingly mediated by residues on the β4 strand, the α4 helix and the extended loop between the α5 and α6 helices, termed as the ‘ATP-lid’ (56), with the previously defined QTK-loop (57) of the transducer domain forming salt bridges with the γ-phosphate of AMP-PNP.

Nevertheless, some unexpected local features were also observed in the structure of pP1192R-ATPase (Supplementary Figure 8B, D). First, pP1192R lacks the extended N-terminus, which forms a short helix (α1) and arches over the ATP-binding site of the opposite protomer created by crystal packing, partially constituting the dimeric interface in the other Topo IIA ATPases. Second, an additional α4’ helix, generated by six amino acids (A108-K113) insertion (insert3) found exclusively in pP1192R, was present in the long connecting loop between the β4 strand and the α5 helix that located at the tip of the pP1192R-ATPase. In addition, a short linkage between the α9 helix and the β11 strand and an extended intervening loop with an extra 3_10_ helix (η1) formed between the β11 and β12 strands were found at the base of the pP1192R-ATPase transducer domain, which may result from the variant starting position of the β11 strand in sequence. This extended intervening η1 helix protrudes towards the η2 helix that adjacent to the eukaryotic insertion region of the opposite protomer, pushing it into a distinct orientation ∼8 Å away from the counterpart observed in the *S. cerevisiae* Topo II structure (Figure 3E). Finally, the K-loop, characterized in the eukaryotic Topo IIAs sequence and proven to be important for DNA strand passage activities, is somewhat conserved in pP1192R and consists of three lysine and one arginine. This region is also disordered in pP1192R (Figure 3C), as previously shown in the structure of human or yeast Topo IIs (56, 57).

### Structure of m-ASMA trapped pP1192R-DNA complex reveals both pre- and post-cleavage states

**Figure 4.**
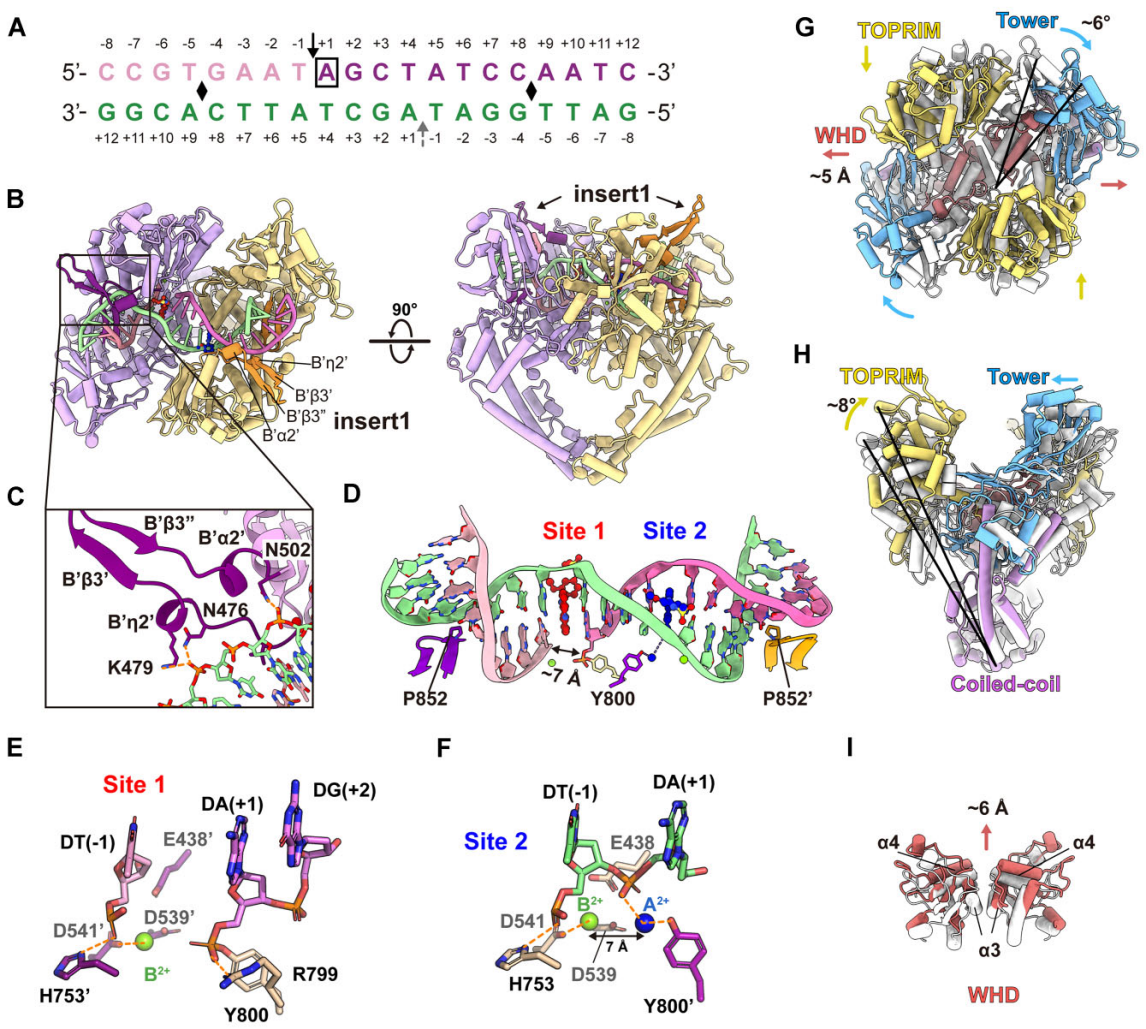
Structure of the pP1192R–DNA–m-AMSA complex. (A) DNA substrate used for structural study. The cleavage sites are indicated by solid black arrow (at Site 1) and dashed grey arrow (at Site 2), respectively. Positive and negative numbers (+1 to +12 and –1 to –8) designate nucleotides downstream and upstream of the scissile phosphate, respectively, with the +1 nucleotide (boxed) at Site 1 forming a phosphotyrosyl linkage with Y800. Black diamonds indicate DNA bend points. (**B**) Orthogonal views of the pP1192R–DNA–m-AMSA complex. One protomer is shown in purple and the other in yellow. The two insert1 regions and the DNA duplex are highlighted. (**C**) Close-up views of the β-hairpin. Key amino acid side chains interacting with DNA are shown as sticks and labeled. Orange dashed lines depict hydrogen bonds. (**D**) Zoomed-in view of the DNA duplex configuration bent by pP1192R. The P852 inserted into the DNA minor groove is shown in stick and the DNA duplex is color as in (A). Two m-AMSA molecules are shown in red (at Site 1) and blue (at Site 1), respectively. Mg^2+^ ions are indicated by green spheres. (E and F) The positions of Mg^2+^ ions at post- (**E**) and pre-cleavage (**F**) sites, illustrating DNA (colored as in (D)), Mg^2+^ ions, catalytic amino acids (E…DxD) and metal coordination (indicated by orange dashed lines). (**G–I**) Structural superimposition of the pP1192R–DNA–m-AMSA complex and the apo structure (the C-gate closed state) reveals the conformational changes in DNA-contacting regions. The individual domains in the pP1192R–DNA–m-AMSA complex structure are colored as in Figure 1, while the apo structure is colored white.

To improve the resolution of the DNA binding/cleavage domain, 3D focused refinement was performed (Supplementary Table 1 and Supplementary Figure 6). Reconstruction of the DNA-binding/cleavage domain were generated at 2.76 Å resolution. The high quality EM map allow us to build the atomic model of the pP1192R DNA-binding/cleavage domain in complex with both DNA duplex and m-AMSA (Figure 4A, B). Secondary structural elements of the protein, all the DNA base pairs, two m-AMSA molecules, and ions (presumably Mg^2+^ as it presents in the buffer) were well resolved (Supplementary Figure 7 and Supplementary Figure 9). The overall configuration of the complex resembles the drug-poisoned cleavage complex of eukaryotic and prokaryotic Topo IIAs (14, 19, 24), with the C-gate adopting a closed conformation (Figure 4B). In particular, the insert1 region, disordered in the apo structures, was clearly identified as a β-hairpin consisting of two antiparallel β-strands, a long intervening loop between the strands and two small helices at both sides (Figure 4B, C and Supplementary Figure 9). It is vertically oriented and points straight up towards the ATPase domain. For further description, we termed them as B’η2’, B’β3’, B’β3’’ and B’α2’, respectively. As presented in other Topo IIA-DNA complexes, the DNA duplex was trapped in the DNA-gate and symmetrically kinked by intercalation of a proline (P852) from each protomer into the DNA minor groove at two points (Figure 4B, D).

**Figure 5.**
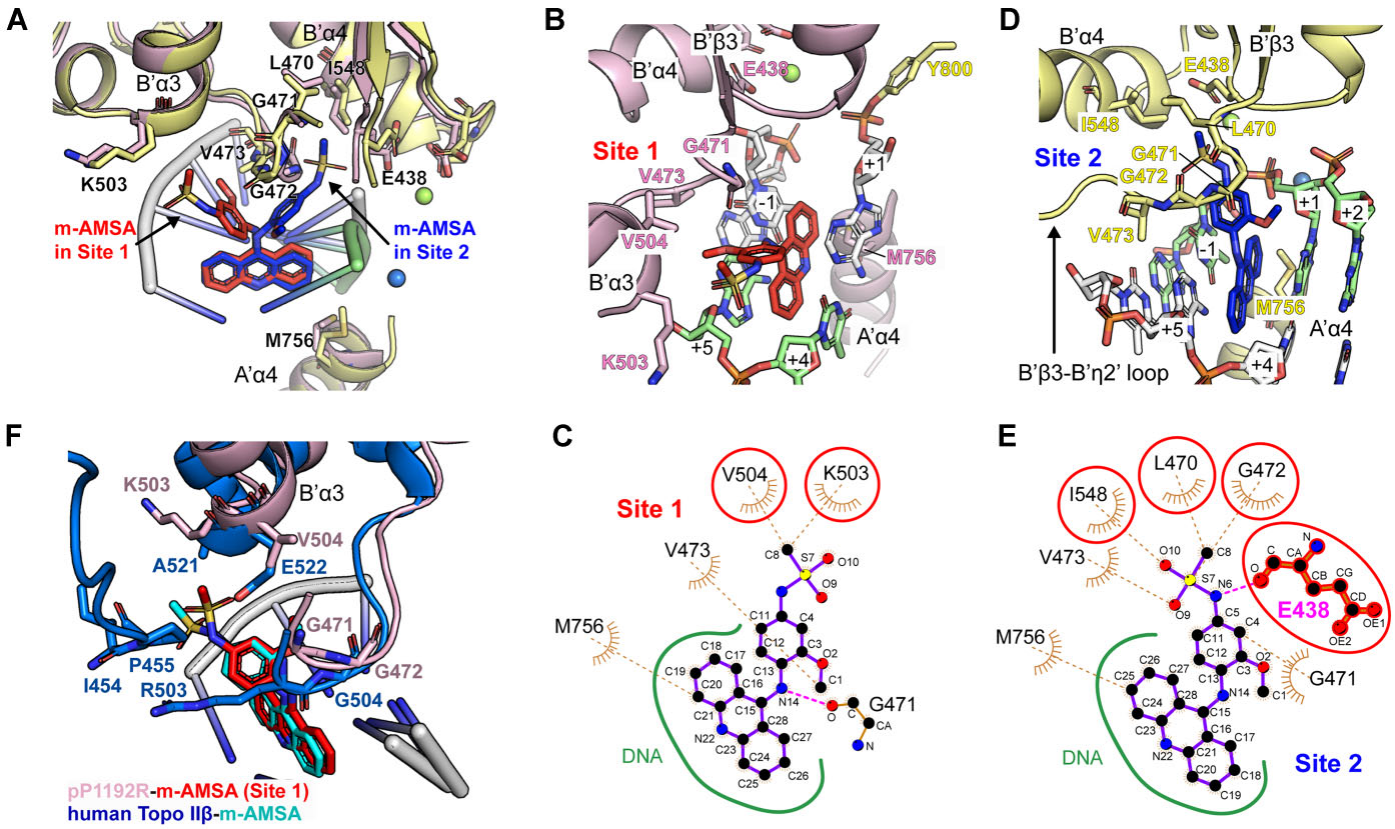
Structural basis for dual modes of pP1192R inhibition by m-AMSA. (**A**) Structural superimposition of Site 1 and Site 2. The two protomers are pink and yellow, with m-AMSA red in Site 1 and blue in Site 2. The key residues involved in m-AMSA–pP1192R interaction are shown as sticks. (**B** and **D**) Detailed view of drug-protein interaction at Site 1 (B) and Site 2 (D). Key residues involved in m-AMSA-pP1192R interaction are shown and labelled. Mg^2+^ ions are depicted as spheres. (C and E) LigPlot analysis of drug-protein interaction at Site 1 (**C**) and Site 2 (**E**). The residue E438, which is involved in hydrogen bonding, is shown as a sphere and a stick, and the amino acids that differ at the two interaction sites are circled in red. (**F**) Structural superimposition of m-AMSA-binding sites in human Topo IIβ (PDB code: 4G0U) and pP1192R (Site 1).

Surprisingly, the EM density clearly supported different cleavage states of the DNA duplex (Figure 4D and Supplementary Figure 9). In one strand, the rupture of the phosphodiester bond was observed between –1 and +1 nucleotides, with the 5′-phosphate of the +1 adenine covalently attached to catalytic tyrosine (Y800) of the cleavage active site, confirming the formation of the cleavage complex, and a m-AMSA molecule was found wedged into the DNA base pair stacks (+1/+4 and –1/+5) flanking the cleaved scissile phosphate. To simplify further description, we designate this post-cleavage site as Site 1. However, there was coherent density in the EM map of the other DNA strand, with no phosphodiester bond break was found at the expected scissile phosphate, indicating a pre-cleavage state (Supplementary Figure 9). One m-AMSA molecule stacked between the DNA base four base pairs away from the Site 1 m-AMSA, albeit with a distinct orientation (Figure 5). Moreover, an extra density appeared bridging the phenolic hydroxyl group of the Y800 and the 5′ phosphate of +1 adenine (Supplementary Figure 9), reminiscent of the position of the metal ion A^2+^ proposed in the intermediate state of the classical two-metal catalysis model. In this intermediate state, the metal ion A^2+^ coordinates with the bridging 5′-oxygen of the scissile phosphate and the nonbridging oxygen atom of the catalytic water or ribose hydroxyl and activates the nucleophilic attack (28). Accordingly, we have proposed the extra density as A^2+^ and refer to this pre-cleavage site as Site 2.

The high-resolution structure reveals the detailed interplay between the protein, the DNA duplex, and the metal ions (Supplementary Table 3 and Supplementary Figure 10). For Site 1, the typical post-cleavage state was present, with the 5′-phosphotyrosyl moiety of the +1 adenine and the 3′-oxyanion group of the –1 thymine separated by ∼7 Å (Figure 4D). A single Mg^2+^ at Site 1 was consistently placed in a position previously defined as B^2+^ in the *S. cerevisiae* Topo II structure (27) and commonly shown in resolved structures of the Topo IIs cleavage complexes (19). In pP1192R, this Mg^2+^ is mainly coordinated with D541, while slightly contacting DT (-1) and the other two residues (E438 and D539) of the E…DxD motif (Supplementary Table 3 and Figure 4E). For Site 2, A^2+^ positions 7 Å distant from the B^2+^ and coordinates both the scissile phosphate and the nucleophilic hydroxyl group of the Y800 (Figure 4F and Supplementary Figure 10).

Outside the ion–protein–DNA interaction sites, extensive protein-DNA interactions are observed mainly between the base pairs ranging from the drug intercalation site to the DNA bending site (base pairs –1/+5, –2/+6, –3/+7 and –4/+8) and the WHD, TOPRIM and Tower domains (Supplementary Table 3 and Supplementary Figure 10A). Most of the secondary structural elements involved in DNA binding are conserved among Topo IIs, with the exception of the pP1192R-specific insert1 region. In this region, potent protein-DNA interactions were found, however, only for residues of the two small helices B’η2’ and B’α2’, while the β-hairpin was not involved in DNA interaction (Figure 4C). The protein-DNA interactions mainly relate to the phosphate and ribose backbone of the DNA, except for the DNA bending site. At this site, although not conserved, P852 of pP1192R continues to play a role that corresponds to the equivalent isoleucine in its homologs: bending DNA by inserting side chains into stacks of DNA base pairs (Supplementary Figure 2).

Comparison of the m-AMSA-poisoned DNA-binding/cleavage domain with the apo structure (the C-gate closed state) revealed relative conformational reorientations of the DNA-contacting region of the two protomers (Figure 4G–I). Upon DNA incorporation, all these regions, the TOPRIM, the Tower and the WHD, underwent a movement to approach the DNA substrate, resulting in a compressed inter-domain distance within protomers and a narrow DNA binding groove. On the other hand, these movements increase the distance between the two protomers (Supplementary Figure 11), which is reflected in the movements of the Tower region away from the adjacent TOPRIM domain of the other protomer, and in the movements of the WHDs away from one another (Figure 4G). Measurements of the distances between the catalytic tyrosine and between the catalytic tyrosine and acidic amino acids of the metal-binding motif (E…DxD) before and after DNA binding more accurately reflect the aforementioned conformational changes in detail and further suggests consistent trend of conformational changes after DNA binding of pP1192R with other Topo IIAs (19) (Supplementary Figure 11).

### Structural basis of pP1192R inhibition by m-AMSA in two distinct modes

As mentioned above, two m-AMSAs were found intercalated between the DNA base pairs (+1/+4 and –1/+5) four base pairs apart in this pP1192R structure, albeit with different orientations (Figure 5A). For both sites, the planar acridine chromophore groups of m-AMSAs intercalate in parallel into the base pairs via van der Waals and π–π interactions with the identical position as previously shown for the m-AMSA-poisoned human Top IIβ structure (25), abolishing the stacking interaction between the base pairs. Although the acridine chromophore group primarily mediates the interactions of m-AMSA with the DNA bases flanking the insertion site, the methanesulfon–m-anisidide group is also involved in drug-DNA interactions, particularly the methoxyaniline moiety therein (Supplementary Table 4).

Further analysis of drug-protein contact network reveals two different models of cleavage site specific binding of m-AMSA to pP1192R (Supplementary Table 4 and Figure 5B–E). At both Site 1 and Site 2, the specific drug–protein interactions mainly involve the bulky methanesulfon–m-anisidide group of m-AMSA, albeit adopting different orientations, while the acridine chromophore group binds exclusively to the side chain of M756 from the A’α4 helix (Figure 5A). At Site 1, where the DNA rupture happened, the methanesulfon–m-anisidide head protrudes in a direction opposite to the DNA break (Figure 5B). This orientation is mainly stabilized by main chain hydrogen bond and a group of van der Waals forces between the methoxyaniline moiety and G471, while few contacts were found between the methanesulfonamide moiety and the K503 and V504 side chains (Figure 5C). Despite a similar orientation, the docking angle of the methanesulfonamide moiety in pP1192R Site 1 was fine-tuned with fewer contacts compared to those in human Topo IIβ (25) (Figure 5F), the original target of m-AMSA. This discrepancy may due to the sequence variability of the drug binding site, as the key residues in stabilizing the drug head moiety in human Topo IIβ, the A521, E522, R503 and P455 (25), are all different in pP1192R (Supplementary Figure 12).

In stark contrast, the methanesulfone-m-anisidide group of m-AMSA at Site 2 pointed to the supposed scissile phosphate, with the methyl head anchored in a hydrophobic pocket formed by L470, G471, G472, and V473 from the connecting loop of the B’β3 strand and the B’η2’ helix (B’β3-B’η2’ loop), and the I548 from B’α4 helix (Figure 5D, E and Supplementary Figure 12). The methoxyaniline moiety was further sandwiched by the B’β3-B’η2’ loop and the DNA strand, and the methanesulfonamide moiety was stabilized by forming hydrogen bonds with DNA-cleavage related E438. In this orientation, extensive interactions were confirmed, with a total of 69 contacts to the protein compared to the 24 contacts identified in Site 1, indicating a more extensive drug-protein interaction at Site 2 (Supplementary Table 4). Since the DNA strand is intact at Site 2 and no m-AMSA orientation consistent with that in Site 2 was found at the site of DNA strand break, we propose that m-AMSA in the Site 2-like orientation may impede the function of pP1192R by preventing DNA cleavage and stabilizing a pre-cleavage enzyme–DNA complex.

## Discussion

Over the past three decades, structural biology has greatly advanced our understanding of how topoisomerases, the fundamental enzymes in many life forms, work and has led to a deep comprehension of drugs that specifically target these enzymes. In this study, we presented the structures of pP1192R, the only known type II topoisomerase expressed by mammalian-infecting viruses, in its different enzymatic stages, comprehensively revealing the structural basis of pP1192R-modulated DNA topology change and drug-protein interaction. Both the large sequence difference of pP1192R and the clearly-shown specific structural features have demonstrated its unique evolutionary position between eukaryotic and prokaryotic Topo II (32). However, the overall architecture of pP1192R, as well as the conformational changes between enzymatic stages, are essentially the same as those of other Topo IIs. This is consistent with the conserved nature of its enzyme function and laterally reflects the functional irreplaceability of topoisomerases.

We have provided the evidence for the inhibitory activity of m-AMSA against pP1192R in the protein-based assay and also against the cell-based ASFV replication. However, the comet assay suggests a dual role for the inhibitory activity of m-AMSA against either pP1192R or host Topo II, and it cannot exclude the possibility of host enzyme activity on the substantial amount of viral DNA present in infected cells. Therefore, a more comprehensive approach using a pP1192R-specific antibody may allow further and more accurate quantification of the m-AMSA-induced covalent attachment of the viral enzyme to DNA. Benefiting from the distinct intercalation orientation of m-ASMA at Site 2 and its blockage of DNA cleavage, we captured the conformation mimicking the snapshot just before or intermediate to the nucleophilic attack. As has been well demonstrated in the classical two-metal mechanism, the A^2+^ must be involved in this step to achieve the activation and stabilization of the catalytic tyrosine. We therefore proposed that the bulk of the extra density at Site 2 could be the A^2+^, which is currently engaged in nucleophile formation, coordinating with both the bridging 5′-oxygen of the scissile phosphate and the nonbridging oxygen atom of the hydroxyl group of Y800. Structural superimposition revealed differences between the A^2+^ position in the current structure and that shown in the structure of *S. cerevisiae* Topo II cleavage complex in a post-cleavage state (27) (Supplementary Figure 10). This difference may result from the positional shift of both DNA substrate and the catalytic tyrosine in the active site before and post the DNA cleavage. Notably, a metal ion with a similar but fine-tuned position to A^2+^ in the *S. cerevisiae* Topo II cleavage complex was found in a drug-free resealed Topo IV-DNA complex of *S. pneumoniae* (58), further suggesting the flexibility of the metal position in the Topo II-mediated the DNA cleavage. Collectively, we proposed a dynamic model for Topo II-mediated the DNA cleavage and provided a better explanation for nucleophile formation. It is also possible that the extra density is provided by the general base, which deprotonates the phenolic hydroxyl group of the catalytic tyrosine. We hope that further studies will provide more evidence for this model by resolving more Topo II-DNA complex structures in various cleavage states.

The complex structure indicated that the m-AMSA acts as a difunctional inhibitor against pP1192R: either trapping the covalent pP1192R-DNA cleavage complex, the conventional mechanism of m-AMSA-mediated poisoning against human Topo II, or stabilizing the non-covalent pP1192R–DNA complex via preventing DNA cleavage. Further structural analysis revealed the interactions between pP1192R E438 and the methanesulfonamide head of the drug when inhibiting the protein in a catalytic inhibitor conformation. This conserved glutamic acid has been proved to be indispensable for Topo II-mediated DNA cleavage (59, 60). Thus, binding of the drug to E438 may disrupt its function in the formation of the pentacovalent intermediate, as elucidated in the two-metal cleavage hypothesis, and thus further reduce the activity of the cleavage. In fact, the co-occurrence of nicked and linear DNA products has previously been observed in fluoroquinolone-induced DNA cleavage by prokaryotic Topo IIs (Supplementary Figure 4) (14) or in etoposide-induced DNA cleavage by human Top2 (61) or gyrases from *E. coli* or *S. aureus* (30). Although the nicked bands in the biochemical data were usually ignored or not discussed, they suggest the possibility that, at least in the above cases, these poisons may also inhibit the corresponding topoisomerases by a combined mechanism. The current complex structure containing DNA substrate with SSB is consistent with and, to our knowledge, provide the first structural basis for the formation of nicked DNA products in the cleavage assay, while the formation of the linear DNA band remains unexplained. Although we were not able to identify the population of particles with DSB-containing DNA substrate when processing the cryo-EM data, increasing the particle number or changing the preparation conditions of the cryo-samples may increase the proportion of complexes with DSB DNA substrates in the samples and further explain the mechanism of linear DNA product formation. Notably, in the recently reported structure of pP1192R in complex with m-ASMA and a originally double nicked DNA substrate (54), only the post-cleavage site (Site 1)-like orientation of m-AMSA was found with a similar drug-protein interaction network around it (Supplementary Figure 12). This finding further supports a preference for m-AMSA to adopt a Site 1-like orientation during post-cleavage intercalation, the conventional mechanism of inhibition.

As to why this novel drug conformation may emerge in pP1192, we speculate it is related to amino acid differences in the drug binding sites of Top IIAs. The intercalation of the polycyclic aglycone core between the base pair stacks of the cleavage site considerably limited the possible binding orientations of m-AMSA. The direction of the methanesulfone–m-anisidide group was thus further determined by binding site-specific interactions with the targets. Due to the nonpolar nature of the methyl head of m-AMSA, its tendency of pointing to hydrophobic pocket shown in the Site 2 were also presented in m-AMSA trapped human Topo II cleavage complex, which is mainly mediated by the P455 and A521. These residues were not conserved in pP1192R and were substituted by charged amino acids D416 and K503, respectively, reducing the interaction strength when m-AMSA binds in this orientation. Reciprocally, the B’β3–B’η2’ loop in pP1192R, which provides the hydrophobic sidewall for the methyl head docking in the Site 2-like orientation, was also not conserved in human Topo II. Substitutions of charged amino acids R503 and K505 abolished the local hydrophobicity of this connecting loop, and may be the determinate for the absence of Site 2-like orientation of m-AMSA in human Topo II (Supplementary Figure 12).

Given the consistency of drug binding site configurations and enzymatic activity modes between pP1192R and its Topo II homologs, it can be concluded that the differences in both mechanism and outcome of inhibitor action are determined by sequence variations around the drug binding site. These discrepancies should be further used to develop virus-specific drugs without interfering with the host cell homologs.

## Supplementary Material

gkae703_Supplemental_File

## Data Availability

The cryo-EM maps have been deposited in the Electron Microscopy Data Bank (EMDB, https://www.ebi.ac.uk/pdbe/emdb/) under accession codes: EMD-39250, EMD-39249, EMD-39245, EMD-39078 and EMD-39077. The atomic coordinates have been deposited in the Protein Data Bank (PDB, https://www.pdb.org) under accession codes: 8YGH, 8YGG, 8YGE and 8YIK. All the accession codes listed here are also shown in Supplementary Tables S1 and Supplementary Tables S2. Source data are provided with this paper. All other data supporting the findings of this study are available within the paper or from the corresponding author upon reasonable request.
