# Correction to ‘Structural basis for difunctional mechanism of m-AMSA against African swine fever virus pP1192R’

**DOI:** 10.1093/nar/gkae771

**Published:** 2024-09-01

This is a correction to: Ruili Liu, Junqing Sun, Lian-Feng Li, Yingxian Cheng, Meilin Li, Lifeng Fu, Su Li, Guorui Peng, Yanjin Wang, Sheng Liu, Xiao Qu, Jiaqi Ran, Xiaomei Li, Erqi Pang, Hua-Ji Qiu, Yanli Wang, Jianxun Qi, Han Wang, George Fu Gao, Structural basis for difunctional mechanism of m-AMSA against African swine fever virus pP1192R, *Nucleic Acids Research*, 2024; https://doi.org/10.1093/nar/gkae703

The graphical abstract had two typographical errors. “Ralexed” should read “Relaxed”; and “lineared” should read “linearized”.

The graphical abstract has been corrected and the published article updated.

This correction does not affect the results, discussion and conclusions presented in the article.

