# Supplementary material for: Structural basis for difunctional mechanism of m-AMSA against African swine fever virus pP1192R
Source: Nucleic Acids Res. 2024 Aug 21;52(18):11301–16. doi: 10.1093/nar/gkae703 (PMC11472052; doi:10.1093/nar/gkae703)
Supplement: gkae703_Supplemental_File [file gkae703_supplemental_file.pdf]

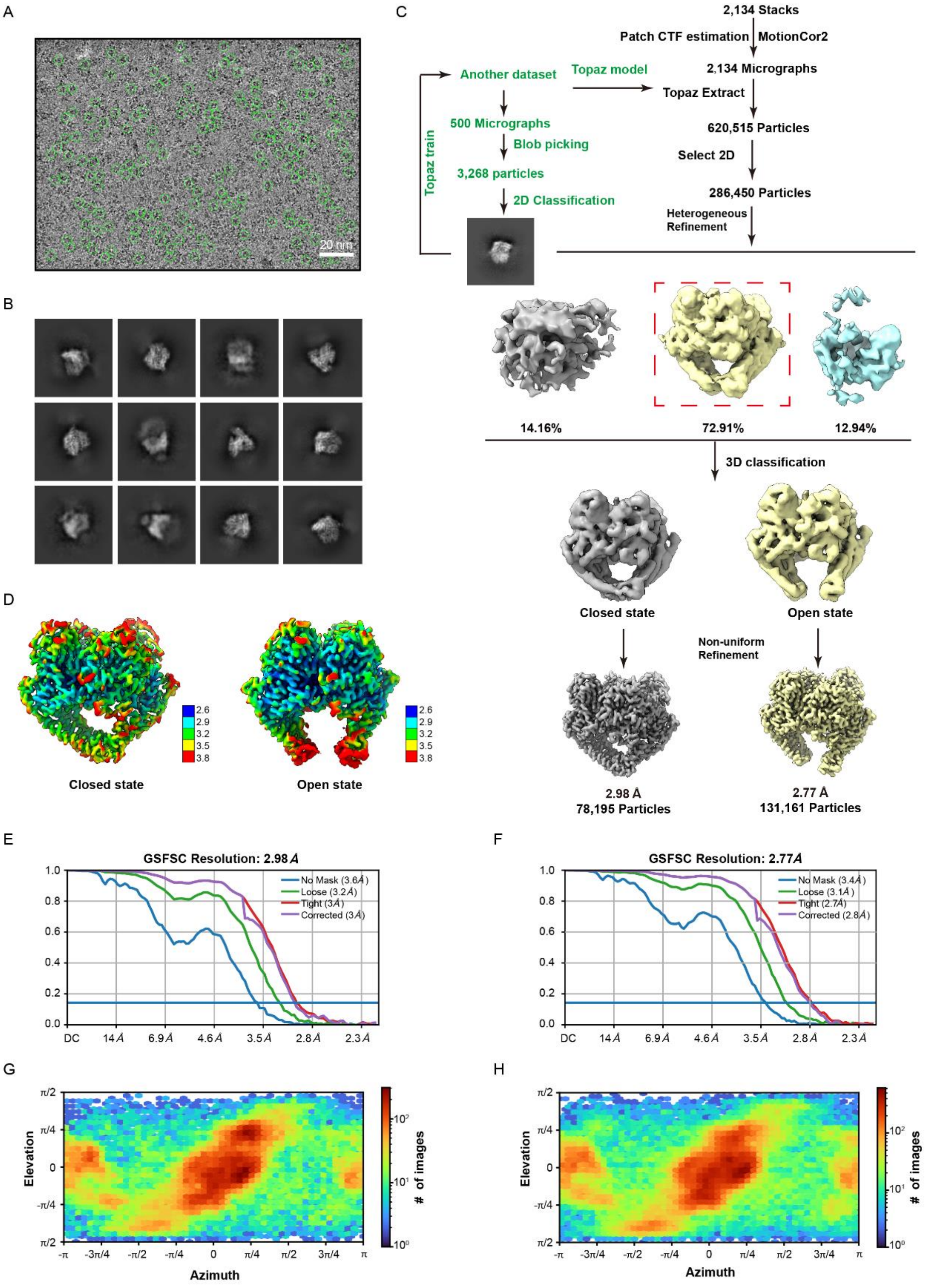


**Supplementary Fig. 1 Cryo-EM data collection, image processing, model building, and refinement.** (A) Representative cryo-EM image with apo pP1192R particles are highlighted by green circles. Scale bar represents 20 nm. (B) Reference-free 2D classification. (C) Flow chart of the cryo-EM data processing. Details can be found in Materials and Methods. (D) Local

resolution for the DNA-binding/cleavage domain in the closed state and open state. (E and F) Golden Standard Fourier Shell Correlation (GSFSC) curve for the DNA-binding/cleavage domain in the closed state (E) and open state (F). (G and H) Euler angle distribution for the DNA-binding/cleavage domain in the closed state (G) and open state (H).

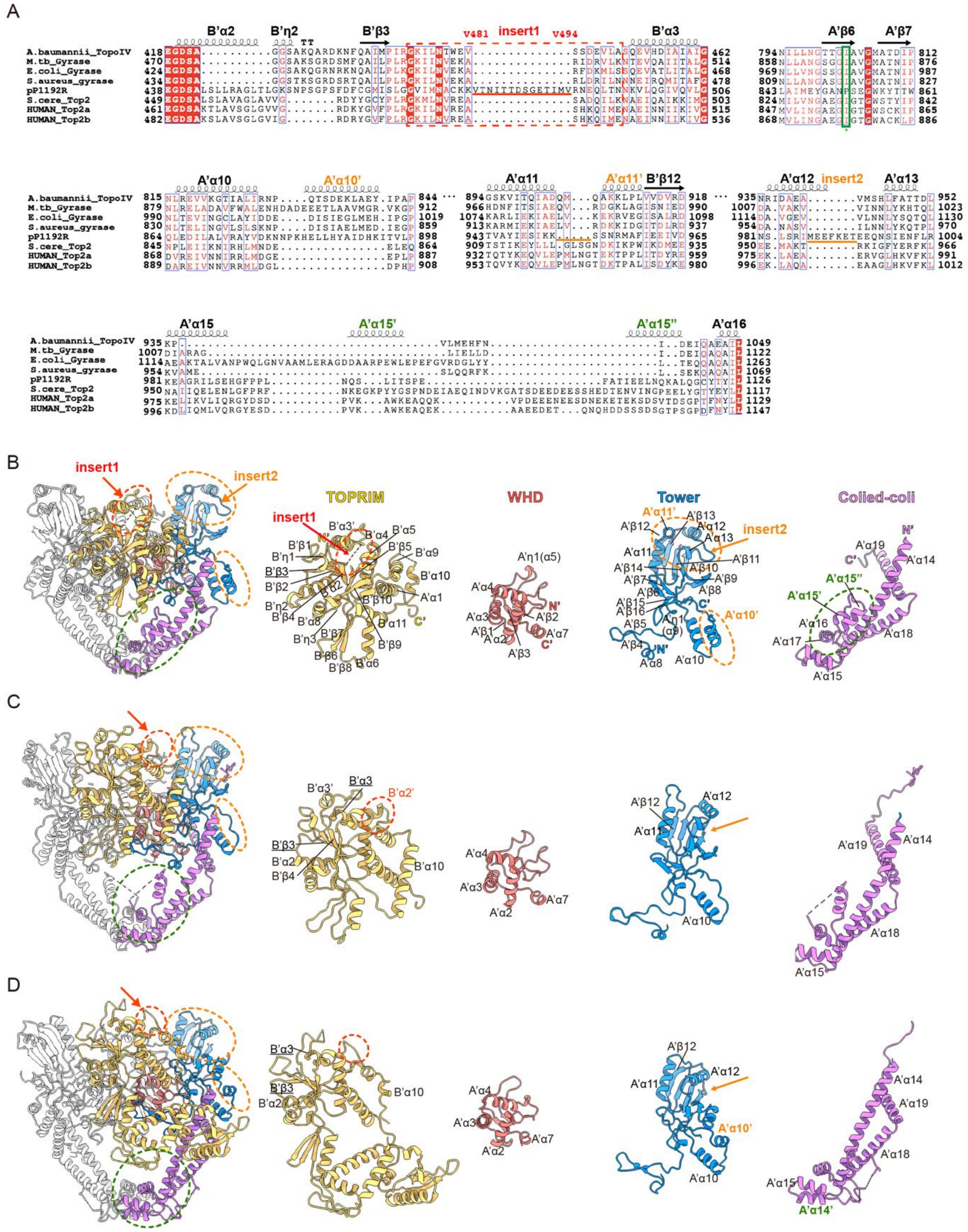


**Supplementary Fig. 2 Structural details of the DNA binding/cleavage domain in pP1192R.** (A) Structure-based sequence alignment of pP1192R DNA binding/cleavage domain with other Topo IIAs homologs. Secondary structural elements of pP1192R are indicated above the sequence alignment. Underline sequences of insert1 and insert2 in red and yellow. G471-N501

is emphasized with a red box. P852 is highlighted with a green box, and an asterisk is noted below the sequence. (B-D) Cartoon representations the closed-state dimers and individual domains of Topo IIAs. In each structural representation, individual domains of ASFV pP1192R (B), human Topo IIα (C), and *E.coli* gyrase (D) are colored as in Fig. 1D.

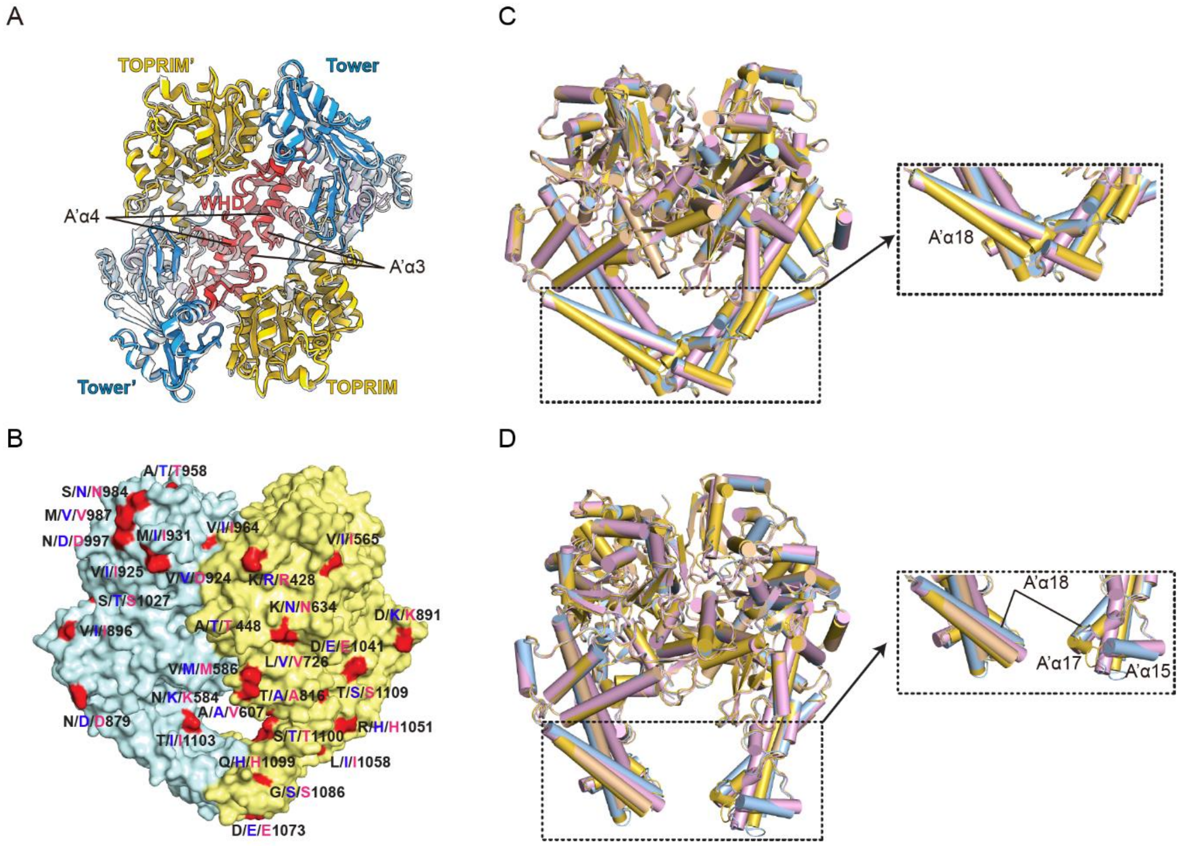


**Supplementary Fig. 3 Comparison between the open and closed states of pP1192R.** (A) Structural superimposition of apo pP1192R in the open state (white) and close state (colored as shown in Fig. 1D). (B) The surface presentation of the DNA binding/cleavage core highlights differing amino acids (shown in red) between ASFV strains. Amino acids of pP1192R from the current ASFV strain (GenBank: AXB50031.1) are labeled in black, while those from strain Georgia07 or strain L60 are shown in blue and purple respectively. One protomer is cyan and the other is yellow. (C and D) Structural superimposition of the closed (C) and open states (D) of the current pP1192R (gold) with those of strain Georgia07 (pink) and strain L60 (conformers IIa and IIIa shown in cyan, conformers IIb and IIIb shown in wheat). Structural variations in the closed states are mainly focused on the C-gate, highlighted by a conformational fine-tuning A' α18, while differences in the open states are observed at A' α15, A' α17 and A' α18.

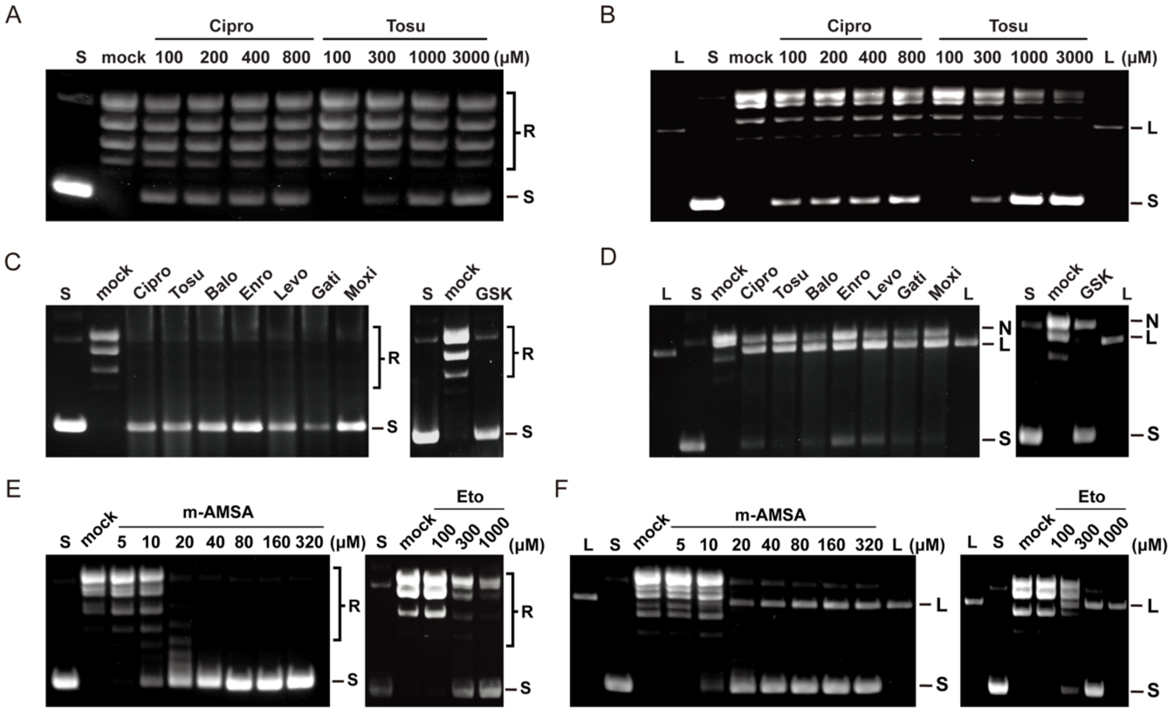


**Supplementary Fig. 4 Evaluation of antibacterial and anticancer drugs against Topo IIs.** (A and B) Inhibition of ciprofloxacin (Cipro) and tosufloxacin (Tosu) against pP1192R evaluated by relaxation assays (A) and cleavage assays (B). Drugs were tested at serial dilutions (final concentrations shown) starting from their respective highest concentration without drug precipitation. S, R and L represent the supercoiled, relaxed and linear DNA bands, respectively. (C and D) Inhibition of fluoroquinolones and GSK299423 against *Acinetobacter baumannii* topoisomerase IV evaluated by relaxation assays (C) and cleavage assays (D). The antibacterial drugs were all tested at a final concentration of 2 μM. The gel shows that fluoroquinolones can induce both single-stranded (linear DNA band) and double-stranded (nicked DNA band) DNA breaks, whereas GSK induces only single-stranded DNA break (linear DNA band). S, R and L represent the supercoiled, relaxed and linear DNA products, respectively. (E and F) Inhibition of anticancer drugs against human Topo IIα evaluated by relaxation assays (E) and cleavage assays (F). The final concentrations of drugs are shown. M-AMSA and etoposide induce only increasing linear products with increasing drug concentration in human Topo IIα, while the nicked DNA bands were at the same level as those shown in the pUC19 plasmid substrate. Linear (L), nicked (N), relaxed (R), and supercoiled (S) forms of DNA are labelled.

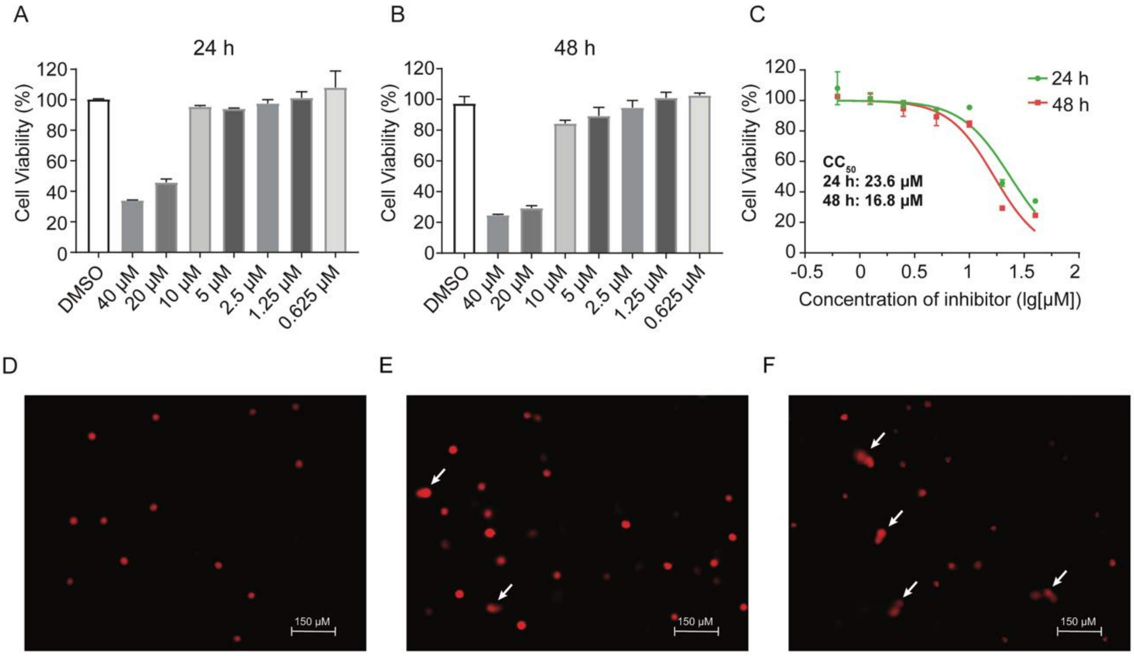


**Supplementary Fig. 5 The influence of m-AMSA on the viability of PAM cells.** (A and B) Assessment of m-AMSA cytotoxicity in PAM cells after 24 h (A) or 48 h (B) of m-AMSA treatment using the CCK-8 assay. (C) Calculation of $CC_{50}$ using nonlinear regression analysis. (D-F) Comet assay analysis of DNA damage in the PAM cells infected with ASFV and not exposed to m-AMSA (D), non-infected and exposed to m-AMSA (E), and infected and exposed to m-AMSA (F). The scale bar in the fluorescence images is 150 μm.

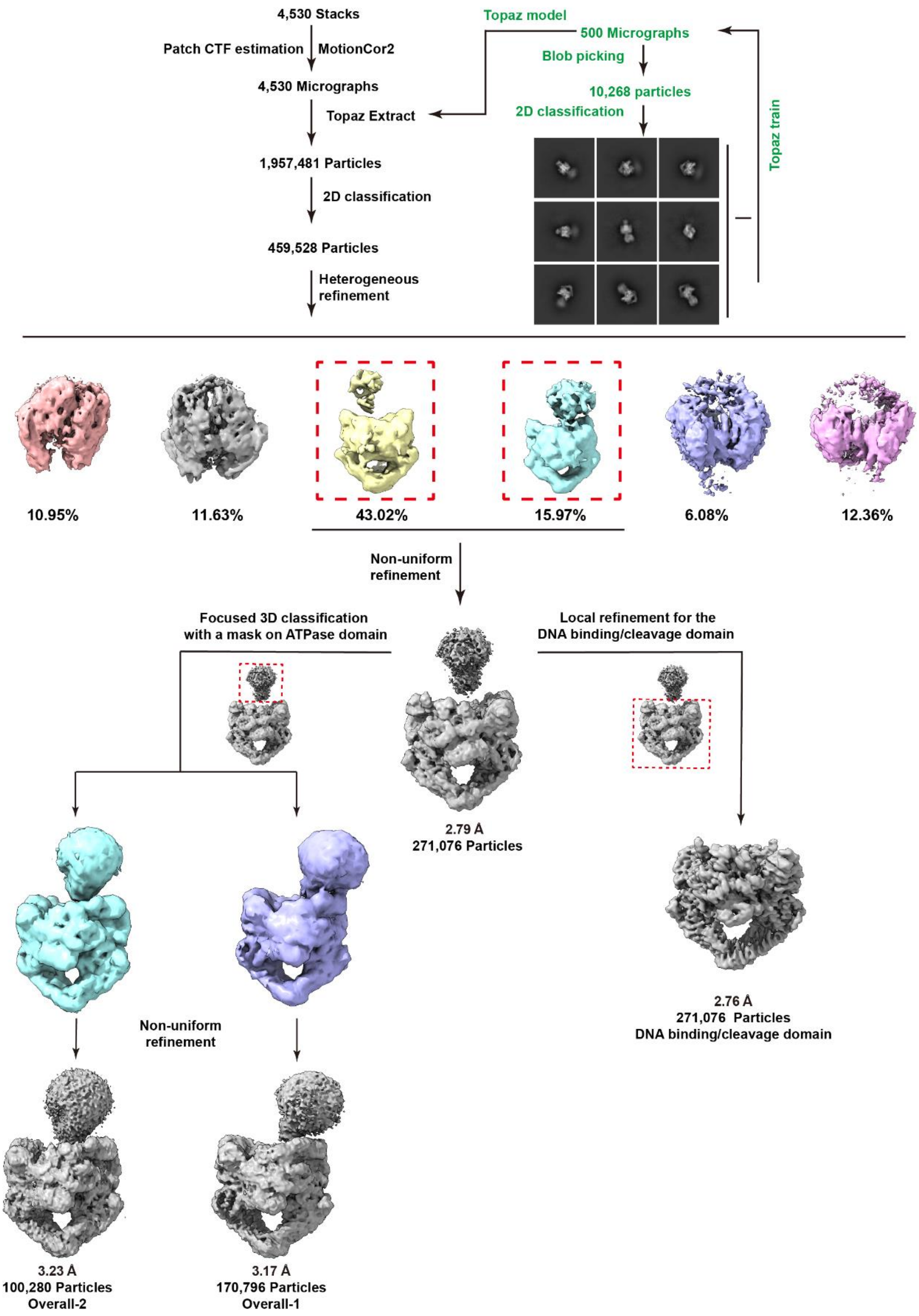


**Supplementary Fig. 6 Flowchart of cryo-EM data collection, image processing, model building, and refinement.** Please refer to the 'Image Processing' in Methods section for details.

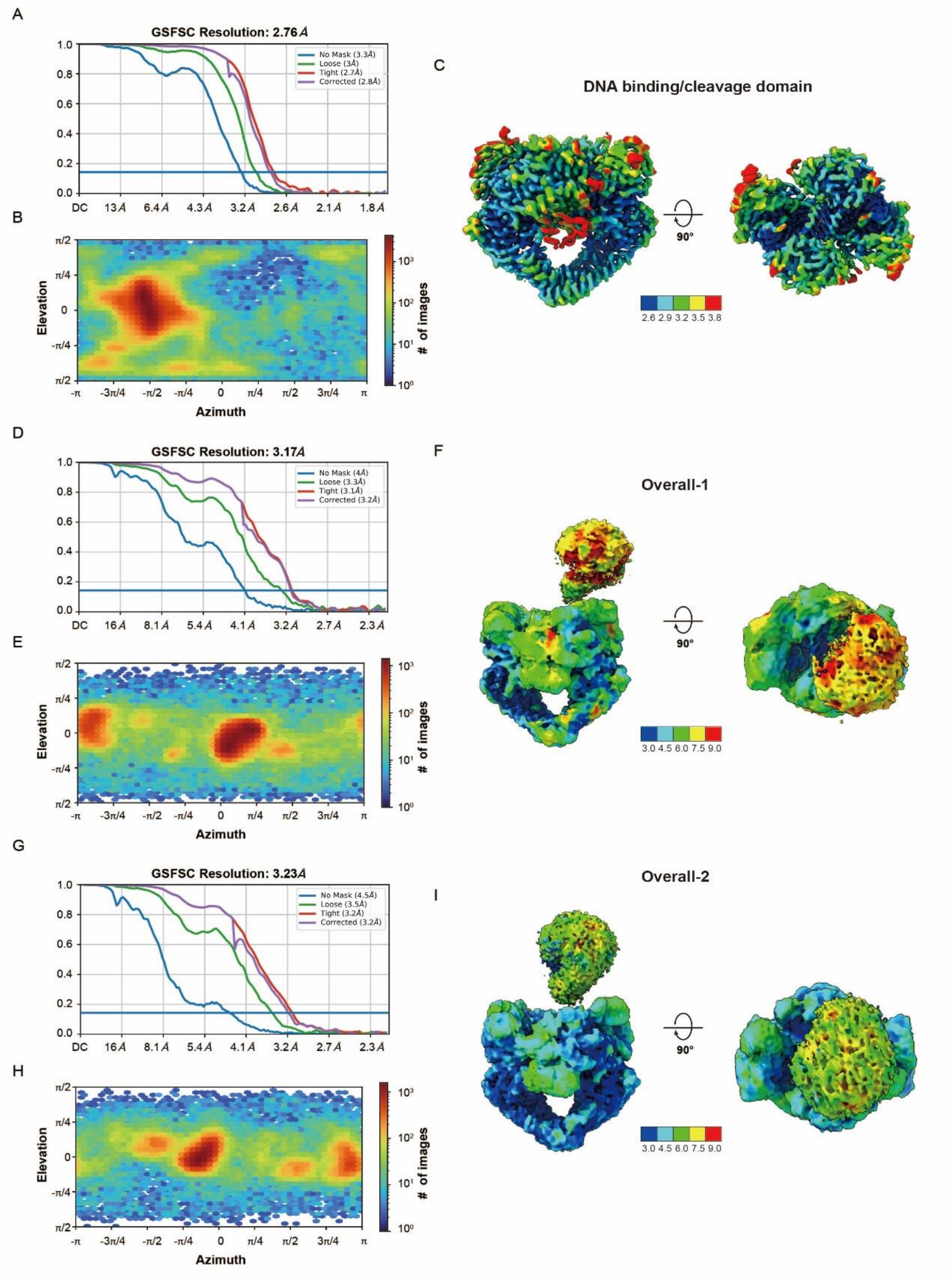


**Supplementary Fig. 7 Structural data validation and local resolution.** The Fourier Shell Correlation (FSC) plot and resolution estimation using the gold-standard 0.143 criterion (A, D, G). Particle angular distribution plots (B, E, H). Local resolution for the DNA-binding/cleavage domain in pP1192R-DNA-m-AMSA complex (C) and for the overall-1 (F) and overall-2 (I).

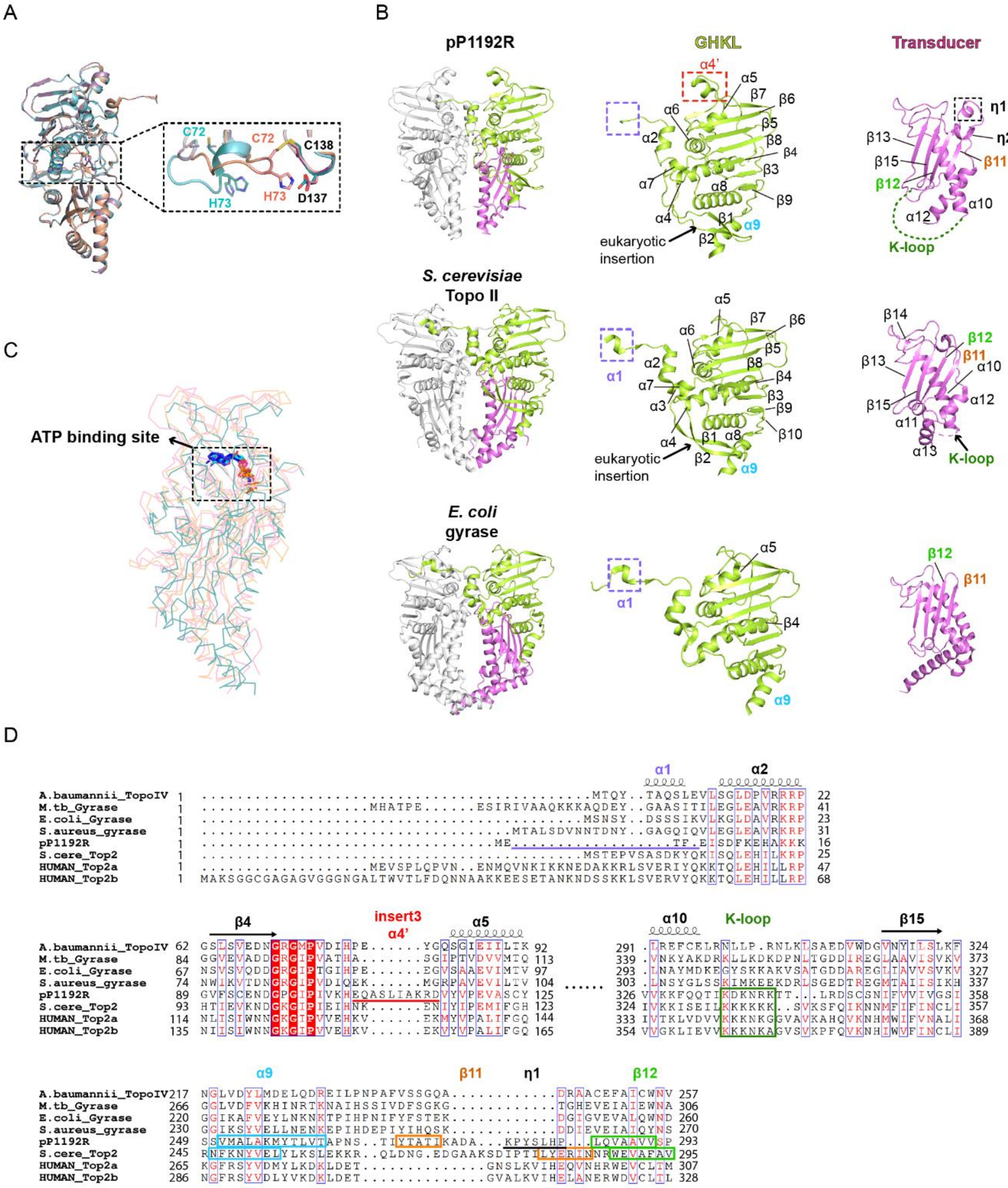


**Supplementary Fig. 8 Structural details of the pP1192R ATPase domain.** (A) Superimposition of ATPase domains in the present pP1192R (purple) with those in the reduced (cyan) and oxidized (orange) forms. The disulfide bond between C72 and C138 has been highlighted on the right, with residues C72, C138, H73, and D137 in stick presentation. (B) Cartoon illustrating the dimers of ATPase domain and individual domains of Top IIAs. (C) Superimposition of ATPase domain monomers in pP1192R (yellow), *S. cerevisiae* Topo II

(pink), and *E.coli* gyrase (cyan). The ATP binding site is indicated by a dashed square. (D) Structure-based sequence alignment of pP1192R ATPase domain with other Topo IIAs homologs. Secondary structural elements of pP1192R are indicated above the sequence alignment. Unique structures in the pP1192R ATPase domain are highlighted.

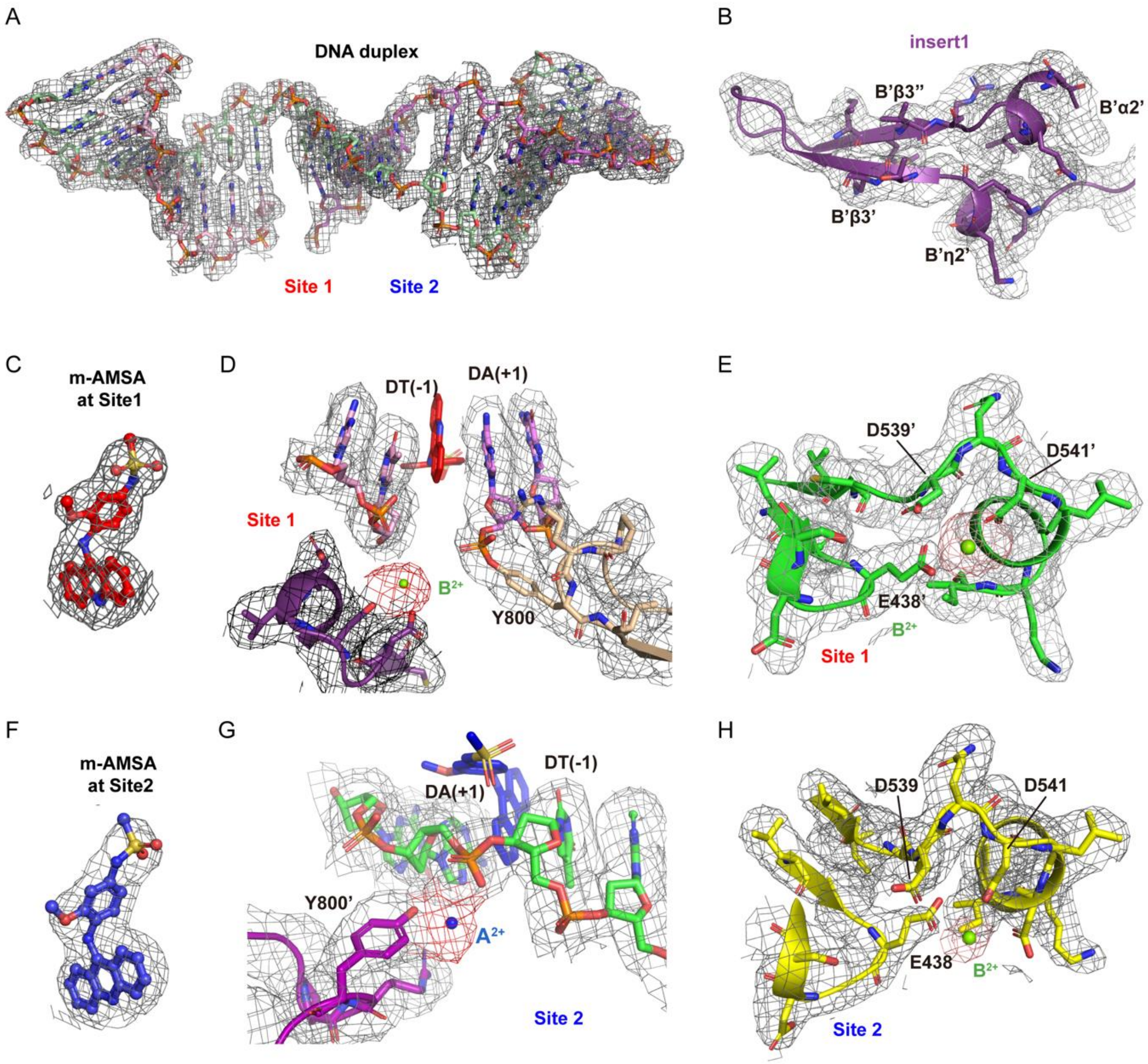


**Supplementary Fig. 9 Cryo-electron microscopy densities and atomic models of critical structural elements.** (A) EM density for different cleavage states of the DNA duplex. (B) EM density displays individual beta strands of insert1. (C) EM density around the m-AMSA at Site 1. (D) Visualization of EM density at cleavage active Site 1, with the +1 nucleotide forming a phosphotyrosyl linkage with Y800. (E) EM density of the metal ion $B^{2+}$ at Site 1, coordinated by the metal-binding motif (E…DxD). (F-H) EM density of the m-AMSA (F), the metal ion $A^{2+}$ (G), and the metal ion $B^{2+}$ together with E…DxD motif (H) at Site 2.

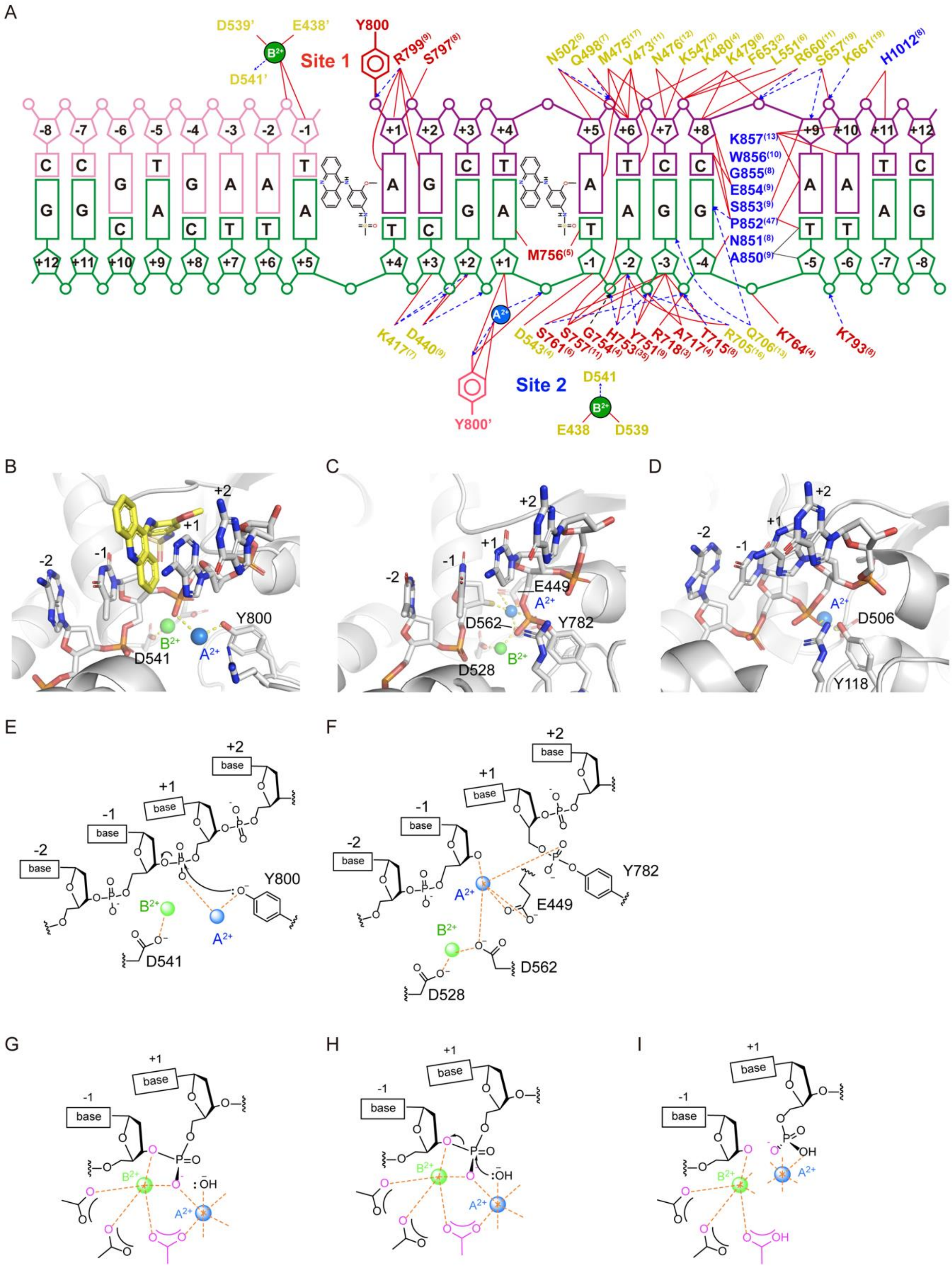


**Supplementary Fig. 10 Detailed interactions between protein, DNA and metal ions.** (A) Schematic of the detailed interactions among the protein, DNA duplex, and $Mg^{2+}$ ions. Red lines highlight all contacts, and the hydrogen bonds or salt bridge interactions are marked by

blue dashed lines. Amino acids in distinct domains are colored as in Fig. 1D. The superscript number indicates the contacts in which the amino acid is involved, and primes indicate another protomer. (B-D) Close-up views showing the metal coordination in the pre-cleavage (Site 2 of the current pP1192R complex) (B), post-cleavage (*S.cerevisiae* Topo II, PDB code 3L4K) (C) and re-sealed (*S. pneumoniae* Topo IV, PDB code 3KSB) states of Topo IIs. The metal coordinations are shown as yellow dashed lines. (E and F) Diagrams of the two metal mechanism in Topo II-mediated DNA cleavage at pre-cleavage (E) and post-cleavage states (F). (G-I) Model of two metal dependent DNA cleavage at pre-cleavage (G), intermediate-cleavage (H) and post-cleavage (I) states, :$\overline{O}$H indicates a deprotonated water molecule or sugar hydroxyl group that has been activated to become a nucleophile.

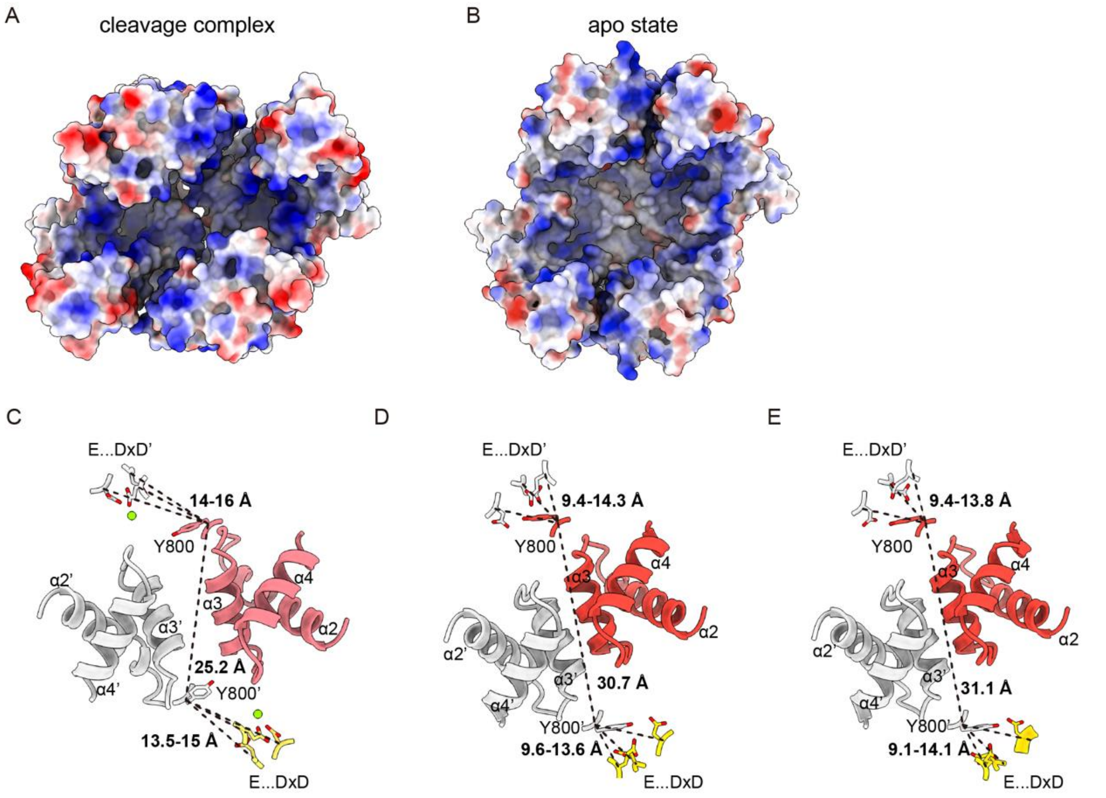


**Supplementary Fig. 11 DNA binding-induced conformational changes.** (A and B) Electrostatic surface presentation of the DNA-binding/cleavage domain in the pP1192R-DNA-m-AMSA complex (A) and apo structure (B). (C-E) Comparison of the the DNA-binding groove in different conformations of pP1192R. In each structure, pP1192R-DNA-m-AMSA complex (C), apo pP1192R in the closed state (D), and apo pP1192R in the open state (E), distinguish one monomer with color and the other with gray, labeling the second monomer with primes. Distances between the two active site tyrosines and between the catalytic tyrosine and acidic amino acids of the metal-binding motif (E…DxD) are also indicated.

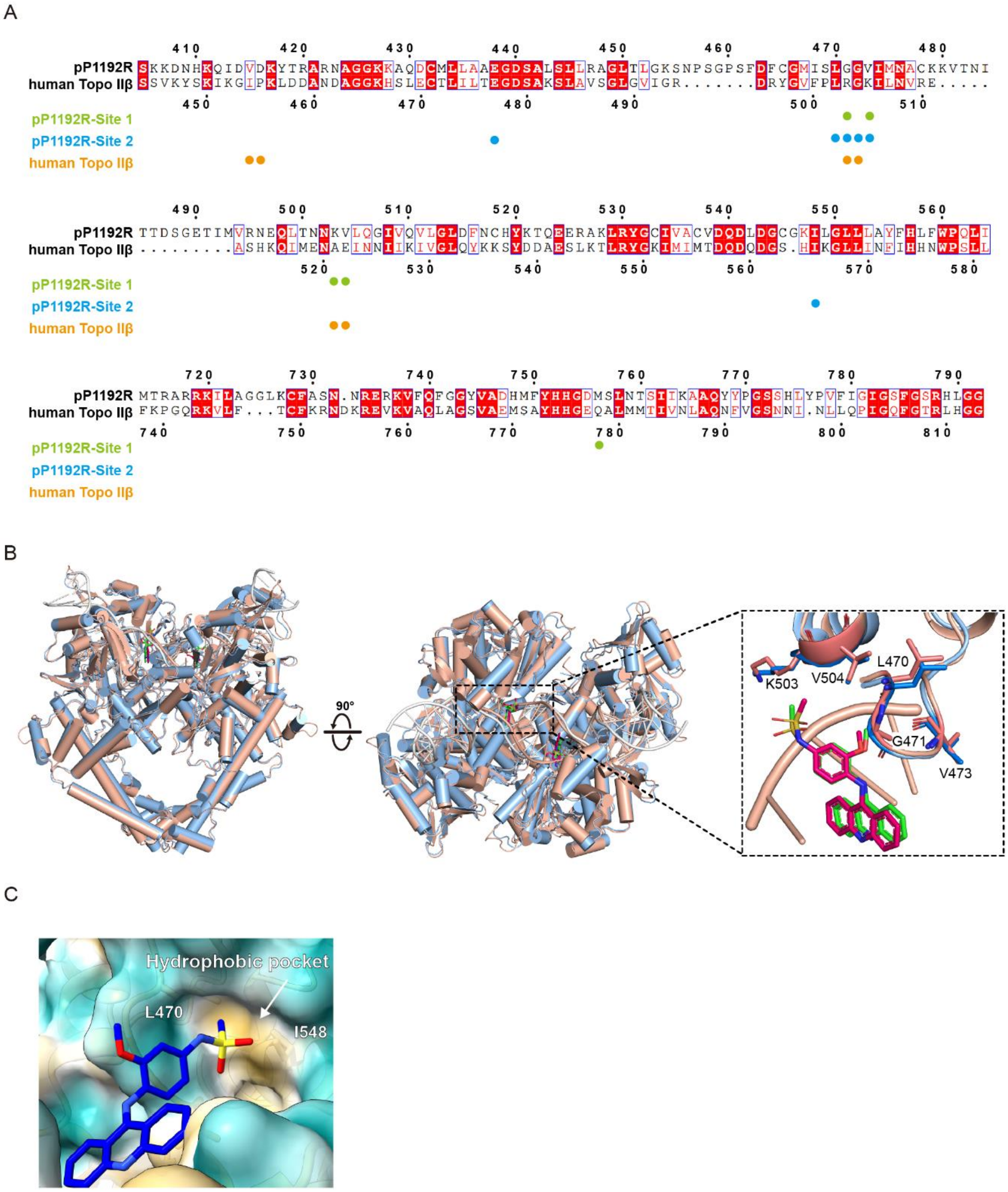


**Supplementary Fig. 12 Structural and sequence analysis of drug binding sites in pP1192R.** (A) Sequence alignment between pP1192R and human Topo IIβ. Colored spheres below the sequences highlight amino acids involved in drug interactions at three distinct drug binding sites. (B) Structural superimposition of the pP1192R-DNA-m-AMSA complex from our study (pink) and the recently reported one (blue, PBD code: 8J9X), with the m-AMSA binding sites and the m-AMSAs inside (shown in red for that from our structure and in cyan for that from

8J9X) highlighted. Key residues for drug-protein interaction are shown as sticks and labelled.

(C) Surface representation of drug binding pocket at Site 2.

**Table S1. Cryo-EM data collection, refinement and validation statistics**

| | pP1192R-apo | | pP1192R-DNA-m-AMSA complex | | |
|---|---|---|---|---|---|
| | Open state | Closed state | DNA binding/cleavage domain | Overall-1 | Overall-2 |
| EMBD | EMD-39250 | EMD-39249 | EMD-39245 | EMD-39078 | EMD-39077 |
| PDB | 8YGH | 8YGG | 8YGE | | |
| **Data collection and processing** | | | | | |
| Magnification | 64 | 64 | 105 | 105 | 105 |
| Voltage (kV) | 300 | 300 | 300 | 300 | 300 |
| Electron exposure (e–/Å$^2$) | 50 | 50 | 50 | 50 | 50 |
| Defocus range (μm) | -1.0 ~ -2.0 | -1.0 ~ -2.0 | -1.0 ~ -2.0 | -1.0 ~ -2.0 | -1.0 ~ -2.0 |
| Pixel size (Å) | 1.08 | 1.08 | 0.85 | 0.85 | 0.85 |
| Symmetry imposed | C1 | C1 | C1 | C1 | C1 |
| Initial particle images (no.) | 620,515 | 620,515 | 1,957,481 | 1,957,481 | 1,957,481 |
| Final particle images (no.) | 131,161 | 78,195 | 271,076 | 100,280 | 170,796 |
| Map resolution (Å) | 2.77 | 2.98 | 2.76 | 3.23 | 3.17 |
| FSC threshold | 0.143 | 0.143 | 0.143 | 0.143 | 0.143 |
| **Refinement** | | | | | |
| Initial model used (PDB code) | 6ZY5 | 6ZY6 | 4GFH | | |
| Model resolution range (Å) | Up to 2.77 | Up to 2.98 | Up to 2.76 | | |
| Map sharpening *B* factor (Å$^2$) | DeepEMhancer (no B factor value) | DeepEMhancer (no B factor value) | DeepEMhancer (no B factor value) | | |

| | | | |
|---|---|---|---|
| Model composition | | | |
| Non-hydrogen atoms | 12246 | 12102 | 13479 |
| Protein residues | 1510 | 1490 | 1558 |
| Nucleotide | \ | \ | 40 |
| Ligands | \ | \ | 5 |
| *B* factors (Å$^2$) | | | |
| Protein | 50.00 | 50.00 | 79.14 |
| Nucleotide | \ | \ | 77.52 |
| Ligand | \ | \ | 126.72 |
| R.m.s. deviations | | | |
| Bond lengths (Å) | 0.004 | 0.004 | 0.005 |
| Bond angles (°) | 1.006 | 0.983 | 0.598 |
| Validation | | | |
| MolProbity score | 2.00 | 1.89 | 1.83 |
| Clashscore | 9.85 | 9.59 | 6.7 |
| Poor rotamers (%) | 0.00 | 0.00 | 1.47 |
| Ramachandran plot | | | |
| Favored (%) | 95.67% | 97.23% | 95.24% |
| Allowed (%) | 4.33% | 2.71% | 4.7 % |
| Disallowed (%) | 0.00% | 0.07% | 0.06% |

**Table S2. Crystallographic data collection and refinement statistics.**

| | pP1192R ATPase domain |
|---|---|
| **PDB ID code** | 8YIK |
| **Data collection** | |
| Space group | P6122 |
| Cell dimensions | |
| *a, b, c* (Å) | 85.75 85.75 211.48 |
| α, β, γ (°) | 90.00 90.00 120.00 |
| Wavelength (Å) | 0.973 |
| Resolution (Å) | 211.5-7.12(1.32-1.30) |
| $R_{merge}$ | 0.092 (1.524) |
| $I / \sigma I$ | 23.900 (2.300) |
| CC1/2 | 1.000 (0.784) |
| Completeness (%) | 98.7 (86.5) |
| Redundancy | 33.4 (14.6) |
| **Refinement** | |
| Limiting resolution (Å) | 1.3 |
| No. reflections | 111384 |
| $R_{work}/R_{free}$ | 0.1871/0.1946 |
| No. atoms | |
| Protein | 3003 |
| Ligand/ion | 31 |
| water | 414 |
| *B*-factors | |
| Protein | 18.77 |
| Ligand/ion | 11.06 |
| Water | 30.51 |
| R.m.s. deviations | |
| Bond lengths (Å) | 0.005 |
| Bond angles (°) | 0.758 |
| Ramachandran plot | |
| Favored (%) | 97.61 |
| Allowed (%) | 2.39 |
| Outliers (%) | 0 |

*Values in parentheses are for highest-resolution shell.

**Table S3. Interactions between DNA duplex, $Mg^{2+}$ ions and pP1192R.**

| DNA chain C | pP1192R$^{chain/Contacts}$ | DNA chain D/E | pP1192R$^{chain/Contacts}$ |
|---|---|---|---|
| DG(-8) | / | DC$^{D}$(-8) | / |
| DA(-7) | / | DC$^{D}$(-7) | / |
| DT(-6) | / | DG$^{D}$(-6) | / |
| DT(-5) | K793$^{A/8}$, A850$^{A/9}$, N851$^{A/3}$, P852$^{A/6}$ | DT$^{D}$(-5) | / |
| DG(-4) | Q706$^{A/5}$, K764$^{A/4}$, N851$^{A/5}$, P852$^{Aaa/11}$ | DG$^{D}$(-4) | Q706$^{B/4}$, K764$^{B/4}$, N851$^{B/10}$, P852$^{B/10}$ |
| DG(-3) | R705$^{A/2}$, Q706$^{A/8}$, T715$^{A/8}$, A717$^{A/3}$, R718$^{A/3}$, Y751$^{A/1}$, S757$^{A/4}$, S761$^{A/6}$ | DA$^{D}$(-3) | R705$^{B/3}$, Q706$^{A/5}$, T715$^{B/6}$, A717$^{B/4}$, R718$^{B/2}$, Y751$^{B/2}$, S757$^{B/4}$, S761$^{B/6}$ |
| DA(-2) | R705$^{A/14}$, A717$^{A/1}$, Y751$^{A/8}$, H753$^{A/19}$, S757$^{A/7}$ | DA$^{D}$(-2) | V473$^{B/1}$, D543$^{B/1}$, R705$^{B/16}$, A717$^{B/1}$, Y751$^{B/7}$, H753$^{B/21}$, G754$^{B/1}$, S757$^{B/7}$, |
| DT(-1) | V473$^{A/1}$, D543$^{A/4}$, H752$^{A/1}$, H753$^{A/16}$, G754$^{A/4}$, M756$^{A/2}$ | DT$^{D}$(-1) | E438$^{B/6}$, G472$^{B/4}$, V473$^{B/6}$, D543$^{B/9}$, I548$^{B/2}$, H752$^{B/1}$, H753$^{B/13}$, G754$^{B/4}$, M756$^{B/2}$, S757$^{B/1}$ |
| DA(+1) | M756$^{A/3}$, Y800$^{B/6}$ | DA$^{E}$(+1) | D440$^{B/1}$, M756$^{B/3}$, R799$^{A/7}$, **Y800$^{A/20}$** |
| DG(+2) | K417$^{A/3}$, G439$^{A/2}$, D440$^{A/8}$, Y800$^{B/1}$ | DG$^{E}$(+2) | K417$^{B/1}$, D440$^{B/10}$, S797$^{A/8}$, R799$^{A/2}$ |
| DC(+3) | K417$^{A/4}$, D440$^{A/1}$ | DC$^{E}$(+3) | K417$^{B/2}$, |
| DT(+4) | / | DT$^{E}$(+4) | / |
| DA(+5) | N502$^{B/1}$ | DA$^{E}$(+5) | V473$^{A/2}$, N502$^{A/1}$ |
| DT(+6) | V473$^{B/3}$, M475$^{B/10}$, Q498$^{B/3}$, N502$^{B/5}$ | DT$^{E}$(+6) | V473$^{A/9}$, M475$^{A/9}$, Q498$^{A/7}$, N502$^{A/4}$, |
| DT(+7) | V473$^{B/1}$, I474$^{B/2}$, M475$^{B/10}$, N476$^{B/13}$, K479$^{B/2}$, K480$^{B/1}$, K547$^{B/8}$ | DC$^{E}$(+7) | I474$^{A/1}$, M475$^{A/8}$, N476$^{A/4}$, K480$^{A/4}$, K547$^{A/2}$ |
| DC(+8) | N476$^{B/4}$, K479$^{B/10}$, F653$^{B/1}$, K699$^{B/1}$, | DC$^{E}$(+8) | N476$^{A/8}$, K479$^{A/6}$, L551$^{A/1}$, F653$^{A/2}$, R660$^{A/1}$, |
| DC(+8) | P852$^{B/9}$, S853$^{B/4}$, E854$^{B/12}$, G855$^{B/1}$ | DC$^{E}$(+8) | Q706$^{A/1}$, P852$^{A/9}$, S853$^{A/7}$, E854$^{A/9}$, G855$^{A/1}$ |

| $Mg^{2+}$ | pP1192R$^{chain/Contacts}$ | DNA$^{chain/Contacts}$ |
|---|---|---|
| chain F-1 | E438$^{B/1}$, D539$^{B/1}$, D541$^{B/2}$ | DT(-1)$^{D/2}$ |
| chain F-2 | E438$^{A/1}$, D539$^{A/1}$, D541$^{A/3}$ | / |
| chain F-3 | Y800$^{B/3}$ | DA(+1)$^{C/6}$ |

| | | | | |
|---|---|---|---|---|
| DA(+9) | K479$^{B/1}$, S657$^{B/8}$, R660$^{B/11}$, P852$^{B/15}$, S853$^{B/3}$, E854$^{B/3}$, G855$^{B/6}$, W856$^{B/7}$, K857$^{B/8}$ | DA$^{E}$(+9) | K479$^{A/2}$, S657$^{A/15}$, R660$^{A/10}$, P852$^{A/19}$, S853$^{A/2}$, G855$^{A/7}$, W856$^{A/10}$, K857$^{A/9}$ | |
| DC(+10) | S657$^{B/3}$, K661$^{B/3}$, K857$^{B/7}$, H1012$^{B/5}$, | DA$^{E}$(+10) | S657$^{A/2}$, K661$^{A/3}$, K857$^{A/6}$, H1012$^{A/6}$ | |
| DG(+11) | H1010$^{B/6}$ | DT$^{E}$(+11) | H1010$^{A/6}$, H1012$^{A/1}$ | |
| DG(+12) | H1010$^{B/1}$ | DC$^{E}$(+12) | H1010$^{A/1}$ | |

*The superscript numbers denote the contacts between pP1192R residues, the DNA duplex, and Mg$^{2+}$ ions, analyzed using the CONTACT program in CCP4 suite with a distance cutoff of 4.5 angstroms. Superscript letters specify the chain to which amino acids, nucleotides, and Mg$^{2+}$ belong. Amino acids on chain B are shaded in gray, while those involved in hydrogen bonding or salt bridges, along with nucleotides, are highlighted in red.

**Table S4. Interaction between AMSA, DNA duplex and pP1192R.**

| m-AMSA | | Site 1 pP1192R$^{chain/Contacts}$ | Site 1 DNA $^{chain/Contact}$ | Site 2 pP1192R$^{chain/Contacts}$ | Site 2 DNA $^{chain/Contact}$ |
|---|---|---|---|---|---|
| Methanesulfonamide moiety 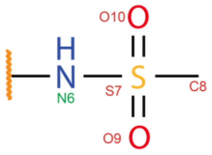  | N6 | / | / | E438$^{A/1}$, G472$^{A/4}$ | DT(-1)$^{C/1}$, DA(+1)$^{C/1}$ |
| | S7 | K503$^{B/1}$ | / | E438$^{A/2}$, G472$^{A/2}$, I548$^{A/1}$ | DT(-1)$^{C/1}$ |
| | C8 | K503$^{B/1}$, V504$^{B/1}$ | / | E438$^{A/4}$, L470$^{A/3}$, G472$^{A/2}$, I548$^{A/1}$ | / |
| | O9 | K503$^{B/1}$ | / | G472$^{A/3}$, V473$^{A/3}$, I548$^{A/1}$ | DT(-1)$^{C/1}$ |
| | O10 | K503$^{B/1}$, V504$^{B/1}$ | DA(+5)$^{C/1}$ | E438$^{A/4}$, I548$^{A/1}$ | DT(-1)$^{C/3}$ |
| Methoxyaniline moiety 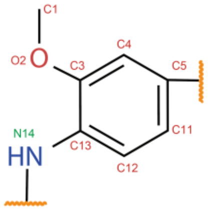  | C1 | G471$^{B/2}$, V473$^{B/1}$ | DA(+5)$^{C/2}$ | G471$^{A/3}$ | DA(+1)$^{C/2}$, DG(+2)$^{C/3}$ |
| | O2 | G471$^{B/2}$ | DA(+5)$^{C/4}$ | G471$^{A/2}$ | DA(+1)$^{C/3}$ |
| | C3 | G471$^{B/3}$ | DA(+5)$^{C/2}$ | G471$^{A/3}$, G472$^{A/2}$ | DA(+1)$^{C/2}$ |
| | C4 | G471$^{B/1}$ | / | G471$^{A/3}$, G472$^{A/2}$ | DA(+1)$^{C/3}$ |
| | C5 | / | / | G471$^{A/1}$, G472$^{A/4}$ | DT(-1)$^{C/1}$, DA(+1)$^{C/1}$ |
| | C11 | / | DT(+4)$^{C/1}$ | G472$^{A/4}$, V473$^{A/1}$ | DT(-1)$^{C/3}$ |
| | C12 | G471$^{B/1}$ | DT(+4)$^{C/1}$, DA(+1)$^{E/3}$ | G472$^{A/4}$, V473$^{A/1}$ | DT(-1)$^{C/3}$, DA(+5)$^{E/1}$ |
| | C13 | G471$^{B/2}$ | DA(+1)$^{E/1}$ | G471$^{A/2}$, G472$^{A/2}$ | DT(-1)$^{C/1}$ |
| | N14 | G471$^{B/2}$ | DA(+5)$^{C/3}$, DA(+1)$^{E/1}$ | / | DA(+1)$^{C/1}$, DA(+5)$^{E/2}$ |
| | C15 | / | DA(+5)$^{C/7}$, DA(+1)$^{E/3}$ | / | DA(+1)$^{C/3}$, DA(+5)$^{E/4}$ |
| Acridine chromophore group | C16 | / | DA(+5)$^{C/4}$, DT(-1)$^{D/3}$, DA(+1)$^{E/5}$ | / | DA(+1)$^{C/2}$, DT(+4)$^{E/1}$, DA(+5)$^{E/7}$ |
| | C17 | / | DT(-1)$^{D/4}$, DA(+1)$^{E/4}$ | / | DT(+4)$^{E/2}$, DA(+5)$^{E/10}$ |

| | | | | | |
|---|---|---|---|---|---|
| 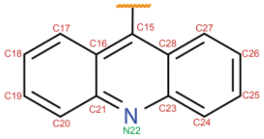 | C18 | / | DT(-1)$^{D/9}$, DA(+1)$^{E/4}$ | / | DT(+4)$^{E/8}$, DA(+5)$^{E/9}$ |
| | C19 | M756$^{B/2}$ | DT(-1)$^{D/8}$, DA(+1)$^{E/4}$ | / | DT(+4)$^{E/11}$, DA(+5)$^{E/7}$ |
| | C20 | M756$^{B/2}$ | DT(-1)$^{D/5}$, DA(+1)$^{E/6}$ | / | DA(+1)$^{C/1}$, DT(+4)$^{E/8}$, DA(+5)$^{E/7}$ |
| | C21 | / | DA(+5)$^{C/3}$, DT(-1)$^{D/3}$, DA(+1)$^{E/7}$ | / | DA(+1)$^{C/4}$, DT(+4)$^{E/4}$, DA(+5)$^{E/7}$ |
| | N22 | / | DT(+4)$^{C/3}$, DA(+5)$^{C/3}$, DT(-1)$^{D/1}$ DA(+1)$^{E/5}$ | / | DT(-1)$^{C/2}$, DA(+1)$^{C/5}$, DT(+4)$^{E/3}$, DA(+5)$^{E/4}$ |
| | C23 | / | DT(+4)$^{C/4}$, DA(+5)$^{C/6}$, DA(+1)$^{E/4}$ | / | DT(-1)$^{C/3}$, DA(+1)$^{C/8}$, DA(+5)$^{E/3}$ |
| | C24 | / | DT(+4)$^{C/8}$, DA(+5)$^{C/5}$, DA(+1)$^{E/1}$ | M756$^{A/1}$ | DT(-1)$^{C/5}$, DA(+1)$^{C/7}$ |
| | C25 | / | DT(+4)$^{C/10}$, DA(+5)$^{C/7}$ | M756$^{A/1}$ | DT(-1)$^{C/8}$, DA(+1)$^{C/8}$ |
| | C26 | / | DT(+4)$^{C/10}$, DA(+5)$^{C/9}$ | M756$^{A/1}$ | DT(-1)$^{C/8}$, DA(+1)$^{C/9}$ |
| | C27 | / | DT(+4)$^{C/4}$, DA(+5)$^{C/10}$ | / | DT(-1)$^{C/6}$, DA(+1)$^{C/7}$ |
| | C28 | / | DT(+4)$^{C/3}$, DA(+5)$^{C/8}$, DA(+1)$^{E/2}$ | / | DT(-1)$^{C/4}$, DA(+1)$^{C/6}$, DA(+5)$^{E/3}$ |

*The superscript numbers denote the contacts between m-AMSA, pP1192R residues, and the DNA duplex, analyzed using the CONTACT program in CCP4 suite with a distance cutoff of 4.5 angstroms. The superscript letters indicate the chain to which the amino acid or nucleotide belongs and the number of contacts in which the amino acid or nucleotide is involved. Amino acids on Chain B are shaded in gray, while those involved in hydrogen bonding or salt bridges, along with nucleotides, are highlighted in red.
